# Dual antiplatelet therapy duration after percutaneous coronary intervention with contemporary drug-eluting stents: A systematic review and network meta-analysis of randomized trials

**DOI:** 10.1371/journal.pone.0357462

**Published:** 2026-09-02

**Authors:** Admire Hlupeni, Mehran Jalilzadehbinazar, Rayvlin J. Liceralde, Hussain A. M. Khan, Ravi Donepudi, Shilpkumar Arora

**Affiliations:** 1 St Luke’s Hospital, Chesterfield, Missouri, United States of America; 2 Midlands State University, Senga, Gweru, Zimbabwe; 3 Division of Cardiovascular, Department of Internal Medicine, Saint Louis University School of Medicine, Missouri, United States of America; Coventry University, UNITED KINGDOM OF GREAT BRITAIN AND NORTHERN IRELAND

## Abstract

**Background:**

Despite widespread adoption of abbreviated (<12 months) dual antiplatelet therapy (DAPT) strategies after percutaneous coronary intervention (PCI), the optimal threshold for DAPT abbreviation remains undefined.

**Methods:**

We conducted a network meta-analysis of randomized controlled trials (RCTs) evaluating various abbreviated DAPT durations in patients undergoing PCI with contemporary drug-eluting stents (DES). We searched PubMed, Embase, and Scopus from January 2008 through March 21, 2026. The efficacy and safety outcomes were major adverse cardiovascular events (MACE) and major bleeding, respectively. Relative treatment effects were estimated using risk ratios (RR) with 95% confidence intervals (CI) using random-effects models. Treatment ranking was assessed using surface under the cumulative ranking curve (SUCRA). The study protocol was registered on PROSPERO (CRD420261349664).

**Findings:**

Twenty-eight RCTs comprising 84,325 patients were included. Compared with 12-month DAPT, abbreviated strategies (1-, 3-, and 6-month) were not associated with a significant difference in MACE (1-month: RR 1.03 [95% CI 0.88–1.22]; 3-month: RR 0.95 [95% CI 0.82–1.10]; 6-month: RR 1.06 [95% CI 0.88–1.27]). In contrast, these shorter durations were associated with significantly reduced major bleeding (1-month: RR 0.59 [95% CI 0.42–0.82]; 3-month: RR 0.64 [95% CI 0.49–0.84]), with a consistent trend for 6-month DAPT (RR 0.68, 95% CI: 0.46–1.00). SUCRA rankings indicated that the 3-month DAPT strategy ranked highest for MACE (lowest ischemic risk); whereas the 1-month strategy ranked highest for major bleeding (lowest bleeding risk), followed by the 3-month strategy.

**Conclusion:**

Shorter DAPT durations (1 & 3 months) were associated with reduced bleeding without evidence of increased ischemic risk compared with the conventional 12-month strategy. Among the abbreviated strategies, the 3-month regimen consistently demonstrated the most favorable overall profile across analyses, suggesting it may represent a pragmatic threshold for DAPT abbreviation. However, these findings should be interpreted cautiously given the absence of statistically significant differences in efficacy and the limitations inherent to study-level network meta-analysis.

## Introduction

The evolution of drug-eluting stents (DES) and potent P2Y12 inhibitors has reshaped the balance between ischemic and bleeding risks following percutaneous coronary intervention (PCI) [1,2]. Historically, prolonged dual antiplatelet therapy (DAPT) of 12 months or longer was recommended to reduce the risk of stent thrombosis and recurrent ischemic events. However, extended DAPT may be associated with an increased risk of morbid and fatal bleeding [1,3]. With improvements in stent design and biocompatibility, several randomized controlled trials (RCTs) have evaluated abbreviated DAPT strategies, including 1-month [4–7] and 3-month regimens [8–11], to reduce bleeding risk while preserving ischemic protection.

Contemporary ACC/AHA and ESC guidelines have integrated this evolving evidence, increasingly endorsing shorter regimens or early transitions to P2Y12 inhibitor monotherapy [12,13]. However, these guidelines primarily rely on pairwise comparisons between individual abbreviated regimens and the conventional 12-month standard, and do not provide direct comparative guidance across multiple abbreviated durations. Consequently, the clinical question has shifted from whether DAPT can be safely shortened to how different abbreviated strategies compare with one another. Moreover, individual trials were often underpowered to detect differences in relatively infrequent ischemic events and frequently relied on composite endpoints, which may obscure the distinct trade-offs between ischemic and bleeding outcomes. As a result, uncertainty persists regarding how specific abbreviated strategies, such as 1-, 3-, or 6-month DAPT, perform relative to each other.

In this context, network meta-analysis enables the integration of direct and indirect evidence, allowing simultaneous comparison and ranking of multiple DAPT strategies within a unified analytical framework. Therefore, we conducted this network meta-analysis to compare the performance of various distinct abbreviated DAPT durations and further clarify the relative balance between ischemic protection and bleeding risk, with the aim of informing more individualized decision-making in contemporary clinical practice.

## Materials and methods

### Study design

This systematic review and network meta-analysis was prospectively registered with PROSPERO (CRD420261349664) [14]. It was conducted and reported in accordance with PRISMA guidelines (S1 Checklist) [15].

### Eligibility criteria

Population: Adult patients (≥18 years) undergoing PCI with second- or third-generation DES. We excluded trials involving atrial fibrillation if most patients required concomitant oral anticoagulation.

Intervention and comparator: We evaluated distinct DAPT durations, including abbreviated (<12 months), standard (12 months), and extended (>12 months) regimens. For presentation of relative treatment effects, the 12-month DAPT duration was used as the reference comparator in both the efficacy and safety networks.

Outcomes: The primary efficacy and safety outcomes were major adverse cardiovascular events (MACE) and major bleeding, respectively, both as defined by the individual trials. Studies were excluded if they had missing clinical outcomes.

Study Design: Eligible studies were parallel-group RCTs comparing at least two distinct DAPT durations, at least one of which was an abbreviated regimen. We excluded non-randomized studies, single-arm or historical-control designs, secondary analyses or post-hoc studies, and trials in which <80% of stents were contemporary.

### Search strategy

We systematically searched PubMed, Embase, and Scopus from January 2008 through March 21, 2026 using a search strategy described in the published protocol [14]. We limited the search to studies published from 2008 onward to reflect the contemporary era of DES, characterized by the widespread adoption of second-generation DES with improved safety profiles and lower rates of stent thrombosis. The search was restricted to peer-reviewed, human-subject clinical trials published in English language.

### Study selection

Study selection was managed in Rayyan, which automatically identified potential duplicates for manual verification by a single investigator. Two reviewers then independently screened titles and abstracts for relevance. Full-text articles of potentially eligible studies were subsequently retrieved and assessed against the inclusion criteria. Any discrepancies were resolved through consensus or by consultation with a third reviewer.

### Data extraction

Two reviewers independently extracted study and patient characteristics, treatment variables and outcomes using a standardized data extraction form. Any disagreements were resolved through consensus. Methodological quality was assessed at the study level using the Cochrane Risk of Bias 2 tool. These appraisals informed the risk-of-bias domain within the GRADE framework, which was used to rate the overall certainty of the evidence.

### Data synthesis

We performed a frequentist network meta-analysis using a multivariate random-effects model implemented in Stata 19.5, College Station, Texas 77845 USA. Model parameters were estimated using the restricted maximum likelihood method. As all included trials were two-arm designs, no adjustment for within-study correlation was required. Relative treatment effects were estimated as risk ratios (RR) with 95% confidence intervals (CI). Forest plots were used to visually display results of individual studies and syntheses.

### Inconsistency assessment

The assumption of consistency was evaluated using a global inconsistency test based on a chi-squared statistic comparing direct and indirect evidence across the networks. Local inconsistency was assessed using node-splitting (side-splitting) analysis to evaluate discrepancies within individual treatment comparisons. Subgroup analysis was used to explore possible causes of inconsistency where relevant. Statistical significance for all inconsistency tests was defined as p < 0.05.

### Treatment ranking

The relative hierarchy of DAPT durations was assessed using surface under the cumulative ranking curve (SUCRA) values, which estimate the probability that each strategy ranks among the most effective or safest options across all treatment durations.

### Publication bias

Potential publication bias and small-study effects were evaluated via visual inspection of comparison-adjusted funnel plots and quantified using a network-wide adaptation of Egger’s precision-weighted linear regression tests. Funnel plots asymmetry was considered statistically significant at a two-tailed p-value of <0.05.

### Certainty of evidence

Certainty of evidence was assessed using the GRADE framework, considering study design, risk of bias, inconsistency, indirectness, and imprecision.

## Results

### Study selection

**Fig 1:**
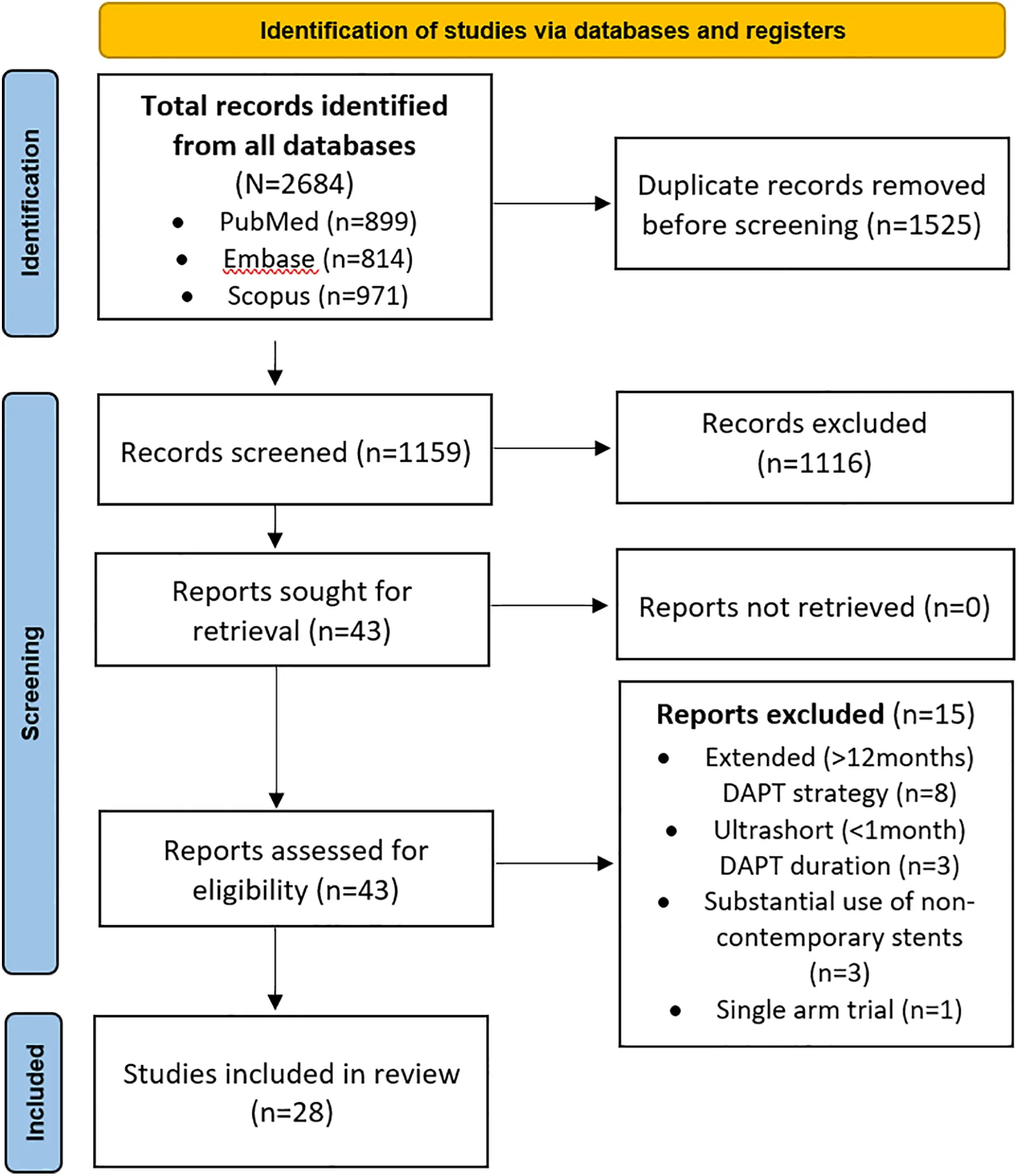
PRISMA flow chart of trial selection. Flow diagram illustrating the study selection process. DAPT = dual antiplatelet therapy; PRISMA = Preferred Reporting Items for Systematic Reviews and Meta-Analyses.

Of 2,684 identified records, 28 trials (84,325 patients) met inclusion criteria [4–11, 16–35] (Fig 1). One trial (HOST-BR) [11] randomized and reported outcomes separately for high and non-high bleeding risk populations; these were included as independent comparisons within the network. Trial characteristics and outcome definitions (MACE and major bleeding) are summarized in Table 1 and S1 Table, respectively. Several high-profile trials were excluded based on predefined criteria, including those evaluating extended (>12 months) [36–43] or ultrashort (<1 month) [44–46] DAPT durations, single-arm designs [47], or inclusion of substantial first-generation stents [48–50] to maintain focus on contemporary abbreviated DAPT strategies.

**Table 1:** Baseline characteristics of included randomized controlled trials according to dual antiplatelet therapy duration.

Trial	DAPT duration compared	DAPT	SAPT after DAPT	N
HOST-BR^a^; Jeehoon Kang, et al. [11]	1m vs 3m	p2y12+asa	p2y12/asa	1598
HOST-BR^b^; Jeehoon Kang, et al. [11]	3m vs 12m	p2y12+asa	p2y12/asa	3299
TARGET DAPT; Hongbo Yang, et al. [16]	3m vs 12m	tica/clop+asa	asa	2445
TARGET-FIRST; G. Tarantini, et al. [7]	1m vs 12m	p2y12+asa	p2y12	1942
4D-ACS; Youngwoo Jang, et al. [35]	1m vs 12m	pras+asa	pras	656
ULTIMATE-DAPT; Zhen Ge, et al. [4]	1m vs 12m	tica+asa	tica	3400
SHARE; Pil-Ki Min, et al. [18]	3m vs 12m	p2y12+asa	tica/clopi	1387
HOST-IDEA; Jung-Kyu Han, et al. [31]	6m vs 12m	p2y12+asa	p2y12/asa	2013
Coroflex ISAR; Uram Jin, et al. [34]	3m vs 6m	p2y12+asa	asa	488
IDEAL-LM^c^; Robert-Jan van Geuns, et al. [30]	4m vs 12m	p2y12+asa	asa	818
STOPDAPT-2 ACS; Hirotoshi Watanabe, et al. [6]	1m vs 12m	pras/clop+asa	clop	4136
MASTER DAPT; M. Valgimigli, et al. [25]	1m vs 6m	p2y12+asa	p2y12/asa	4579
One-month DAPT^d^; Sung-Jin Hong, et al. [23]	1m vs 12m	clop+asa	asa	3020
TICO; Byeong-Keuk Kim, et al. [10]	3m vs 12m	tica+asa	tica	3056
REDUCE; Giuseppe De Luca, et al. [20]	3m vs 12m	pras/tica+asa	p2y12/asa	1496
TWILIGHT; R. Mehran, et al. [10]	3m vs 12m	tica/asa	tica	7119
SMART-CHOICE; Joo-Yong Hahn, et al. [8]	3m vs 12m	p2y12+asa	p2y12	2993
STOPDAPT-2; Hirotoshi Watanabe, et al. [5]	1m vs 12m	clop+asa	clop	3009
DAPT-STEMI; Elvin Kedhi, et al. [33]	6m vs 12m	p2y12+asa	asa	870
GLOBAL LEADERS; Pascal Vranckx, et al. [32]	1m vs 12m	tica+asa	tica	15968
OPTIMA-C; Byoung-Kwon Lee, et al. [22]	6m vs 12m	clop+asa	asa	1367
SMART-DATE; Joo-Yong Hahn, et al. [17]	6m vs 12m	p2y12+asa	asa	2712
NIPPON; Masato Nakamura, et al. [24]	6m vs 18m	p2y12+asa	asa	3307
I-LOVE-IT 2; Jing Qi, et al. [29]	6m vs 12m	clop+asa	asa	907
IVUS-XPL; Sung-Jin Hong, et al. [26]	6m vs 12m	clop+asa	asa	1400
ITALIC^d^; Martine Gilard, et al. [27]	6m vs 24m	p2y12+asa	asa	1822
ISAR-SAFE; Stefanie Schulz-Schupke, et al. [28]	6m vs 12m	clop+asa	asa	4000
SECURITY^d^; Antonio Colombo, et al. [19]	6m vs 12m	p2y12+asa	asa	1399
OPTIMIZE; Fausto Feres, et al. [21]	3m vs 12m	clop+asa	asa	3119

In the HOST-BR trial, high and non-high bleeding risk populations were analyzed as independent comparisons within the network. Continuous variables are presented as mean/median values, and categorical variables as percentages. Acute coronary syndrome (ACS) reflects the proportion of patients presenting with ACS at baseline.

ACS = acute coronary syndrome; asa = aspirin; clop = clopidogrel; DAPT = dual antiplatelet therapy; m = months; n/a = not applicable; N = number of patients; P2Y12=P2Y12 inhibitor; pras = prasugrel; SAPT = single antiplatelet therapy; tica = ticagrelor.

^a^High bleeding risk population.

^b^Non-high bleeding risk population.

^c^Included as 3 months vs 12 months nodes in the analytical networks.

^d^Excluded from the major adverse cardiovascular events (MACE) analysis because MACE was not reported as a standalone endpoint.

### Quality assessment for included trials

**Fig 2:**
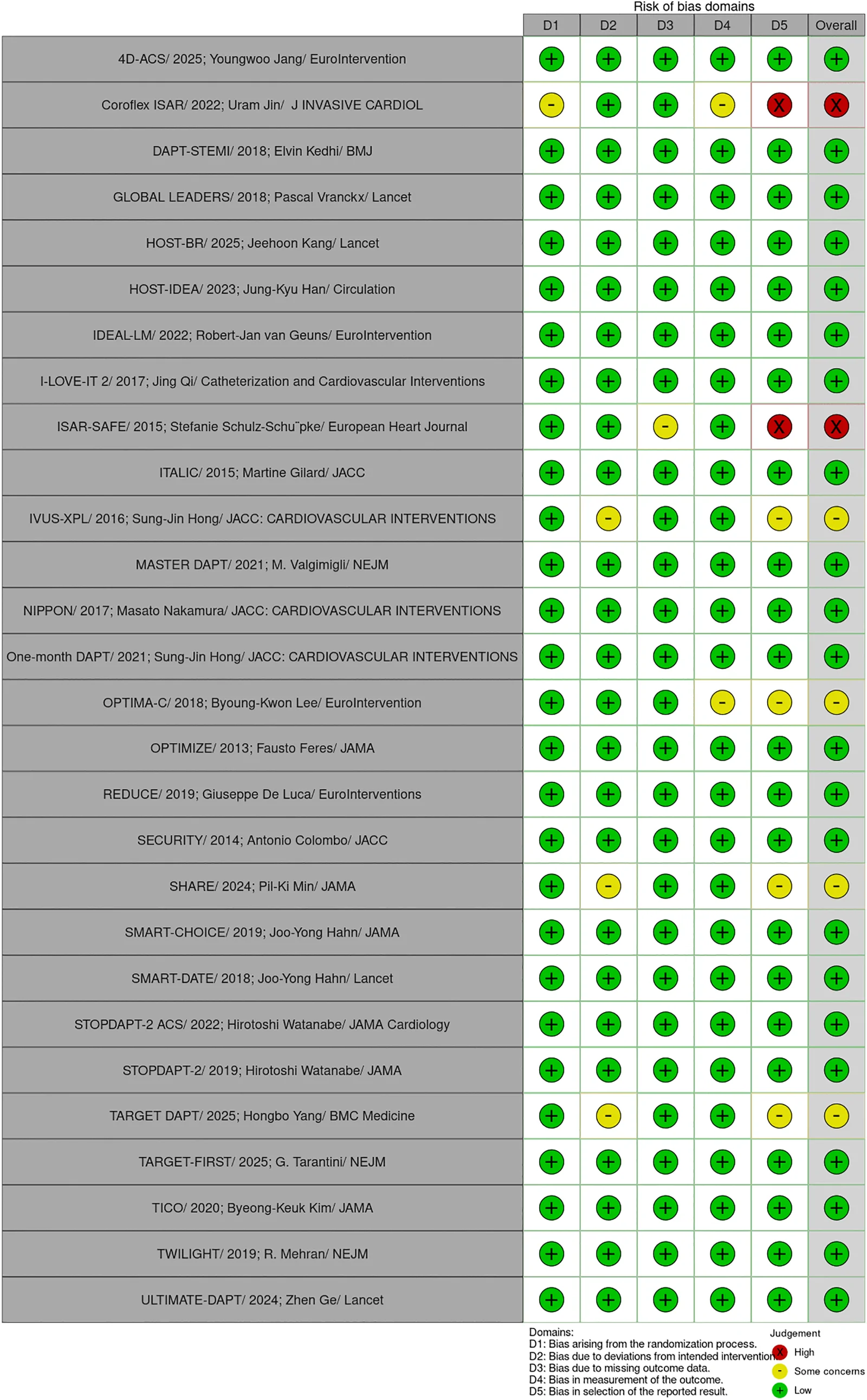
Summary of risk of bias across included randomized trials. Risk of bias for each included randomized trial was evaluated using the Cochrane RoB 2 tool across five domains (D1 to D5). Domain-specific and overall judgments are displayed for each study. Green (+) indicates low risk, yellow (-) indicates some concerns, and red (x) indicates high risk of bias.

Using the Cochrane RoB 2 tool, most trials were judged to be at low risk of bias across all domains, reflecting high methodological quality (Fig 2). A few studies had some concerns, mainly due to post-randomization treatment variability, but these were unlikely to affect objective outcomes. Only a small number of trials were at high risk of bias, primarily related to selective reporting, and these did not materially influence the overall findings. Overall, the evidence base was considered robust with a low risk of bias.

### Efficacy outcome: MACE

**Fig 3:**
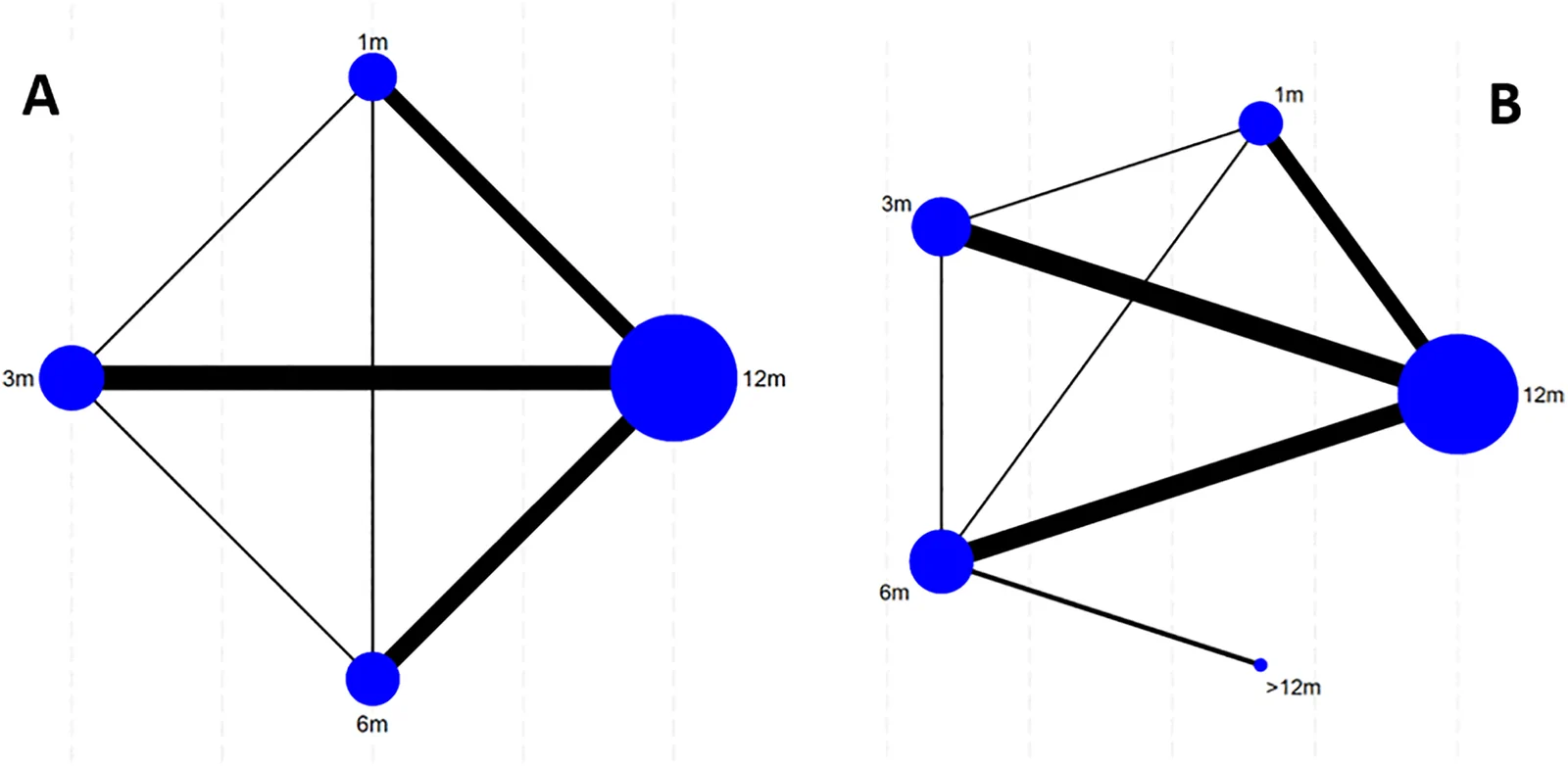
Network maps of dual antiplatelet therapy duration for major adverse cardiovascular events and major bleeding. Each node represents a dual antiplatelet therapy (DAPT) duration, and node size is proportional to the number of patients assigned to that treatment across included trials. Connecting lines indicate direct comparisons between treatment strategies. Line thickness reflects the number of trials contributing to each direct comparison. In panel A (major adverse cardiovascular events), the network includes four DAPT duration nodes (1 month, 3 months, 6 months, and 12 months). In panel B (major bleeding), an additional node (>12 months) is included based on available data from trials reporting bleeding outcomes. m = months.

Of the 28 trials, 24 were included in the MACE analysis, as four trials did not report MACE as a standalone endpoint [19,23,24,27]. Because two of these excluded trials [24,27] evaluated the extended DAPT, the > 12 months duration node was absent from the MACE network. The efficacy network therefore comprised four DAPT duration nodes (1-, 3-, 6-, and 12-month), with the 12-month DAPT used as the reference comparator (Fig 3).

The global inconsistency test indicated marginal inconsistency within the network (p = 0.05), with local side-splitting analysis identifying discrepancies in comparisons involving the 3-month DAPT strategy (12m vs. 3m, p = 0.01; 1m vs. 3m, p = 0.007) (S2 Table). However, subgroup analyses stratified by study setting demonstrated attenuation of the previously observed inconsistency, with no significant global inconsistency detected among multicountry trials (p = 0.94) or single-country trials (p = 0.34).

**Fig 4:**
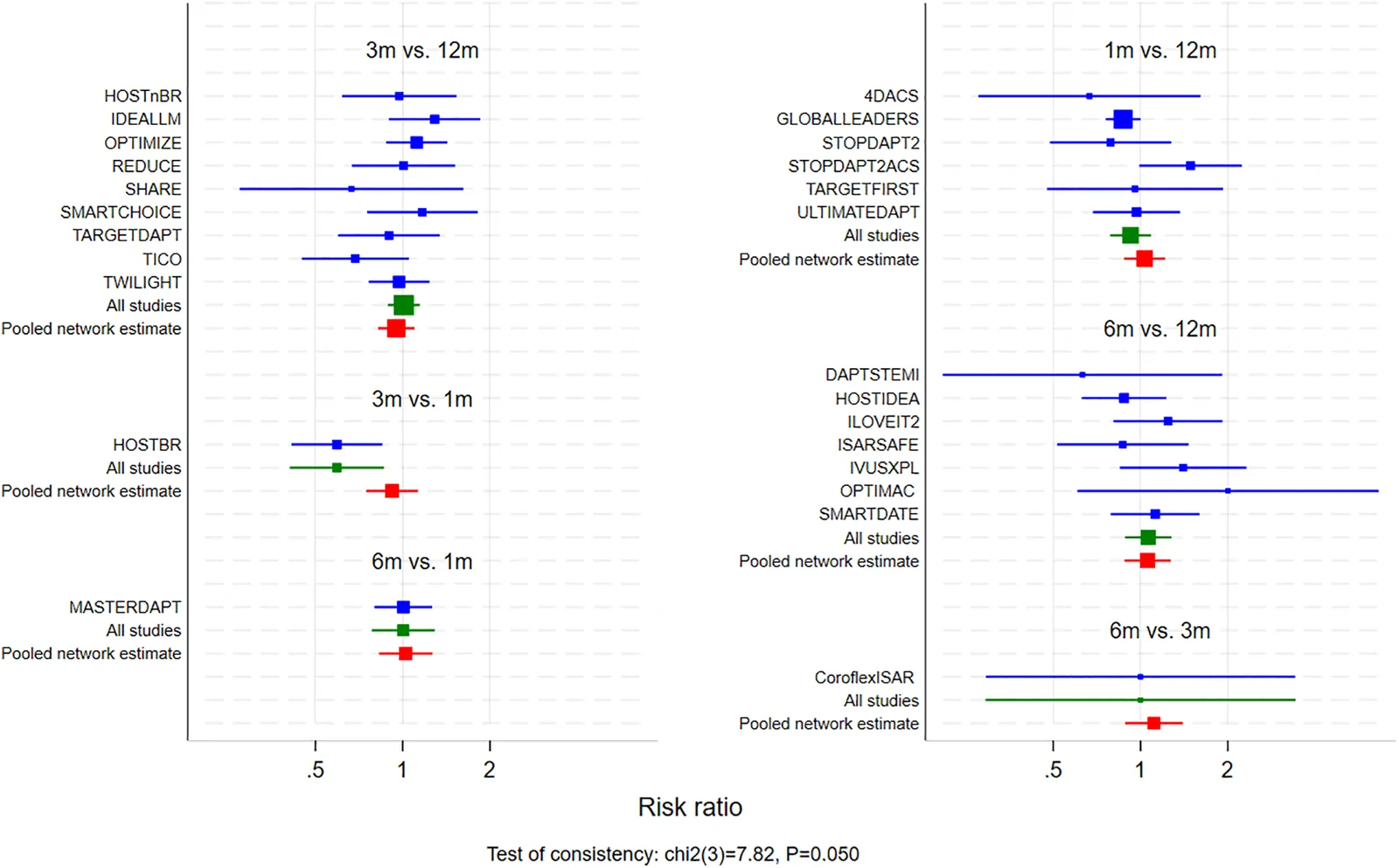
Forest plots comparing dual antiplatelet therapy durations for major adverse cardiovascular events. Forest plots show pairwise comparisons of dual antiplatelet therapy (DAPT) durations for major adverse cardiovascular events derived from the network meta-analysis. Blue squares represent individual trial estimates with corresponding 95% confidence intervals (horizontal lines). Green squares represent pooled direct estimates from conventional meta-analyses, and red squares represent pooled network estimates. The size of the squares is proportional to study weight. Risk ratios (RRs) are presented on a logarithmic scale; values <1 or >1 should be interpreted based on the direction of the comparison. The vertical reference line indicates no difference (RR = 1). m = months.

**Fig 5:**
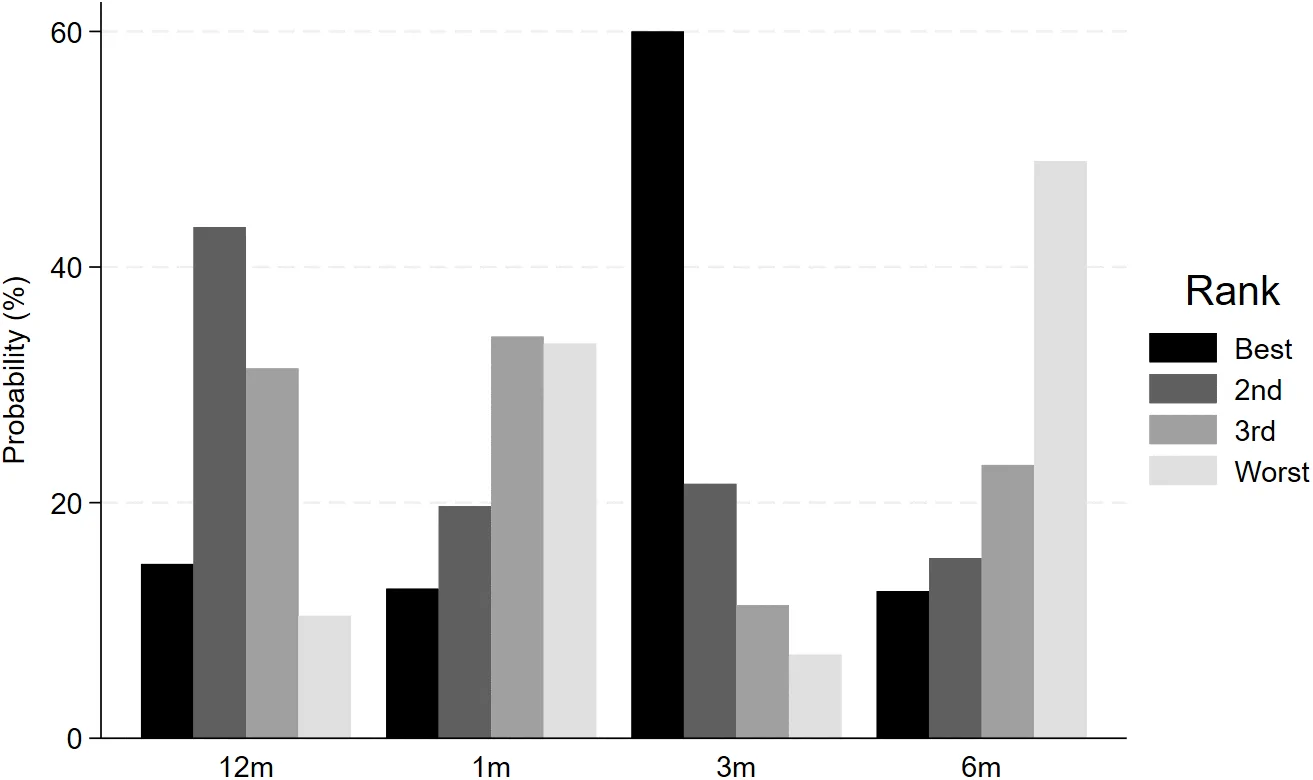
Surface Under the Cumulative Ranking Curve (SUCRA)- based ranking probabilities of dual antiplatelet therapy durations for major adverse cardiovascular events. Bar chart showing the probability of each dual antiplatelet therapy (DAPT) duration strategy ranking at each position (best, second, third, or worst) for major adverse cardiovascular events (MACE), based on the network meta-analysis. Ranking probabilities were derived from the relative treatment effects across the network using a frequentist framework. Each bar represents the probability of a given DAPT duration (1 month, 3 months, 6 months, and 12 months) being ranked at a specific position. The “best” rank indicates the highest probability of achieving the most favorable outcome (lowest risk of MACE), whereas the “worst” rank indicates the least favorable outcome. m = months.

After synthesizing direct and indirect evidence, no DAPT duration significantly differed from the 12-month reference regarding MACE: 1m vs. 12m (RR 1.03; 95% CI: 0.88–1.22), 3m vs. 12m (RR 0.95; 95% CI: 0.82–1.10), and 6m vs. 12m (RR 1.06; 95% CI: 0.88–1.27). Effect estimates were close to unity with confidence intervals spanning the null (Fig 4). Despite these non-significant differences, SUCRA ranking analysis suggested the 3-month strategy had the highest probability of being the most effective (60.0%), while the 6-month duration was most likely to be the least effective (49.0%; Fig 5).

Sensitivity analyses demonstrated consistent findings across clinically relevant subgroups and endpoint definitions (S3 Table and S2 Fig). In trials enrolling exclusively patients with acute coronary syndrome (ACS; n = 8), no abbreviated DAPT duration was associated with a statistically significant difference in MACE compared to the 12-month duration, with 3-month regimen ranking highest (64.3%) and 12-month ranking lowest (7.3%). Similarly, in non-ACS-exclusive trials (n = 16), effect estimates remained non-significant, with 3-month DAPT again ranking highest (39.3%) and 6-month ranking lowest (13.9%), although with greater uncertainty in ranking.

In the network of trials reporting composite (non-hard) MACE definitions that included stent thrombosis and revascularization (n = 20), findings were consistent, with effect estimates close to unity and 3-month DAPT demonstrating the highest probability of being most effective (61.1%), while 1-month ranked lowest (4.1%). In contrast, sensitivity analysis restricted to trials reporting standardized hard ischemic endpoints (death, myocardial infarction, and stroke; n = 4) yielded less precise estimates and variable ranking patterns, with 1-month DAPT ranking highest (64.0%) and 3-month lowest (11.7%); however, these findings should be interpreted cautiously given the limited number of studies and associated imprecision. Overall, these analyses support the robustness of the primary findings across varying clinical populations and MACE endpoint definitions.

### Safety outcome: Major bleeding

All 28 trials reported major bleeding as a standalone outcome and were included in the safety analysis. The safety network included five DAPT treatment duration nodes: 1-month, 3-month, 6-month, 12-month (reference), and >12-month (Fig 3). The bleeding network demonstrated strong consistency, with no evidence of global inconsistency (p = 0.74). Local side-splitting analyses did not identify any significant discrepancies between direct and indirect estimates.

**Fig 6:**
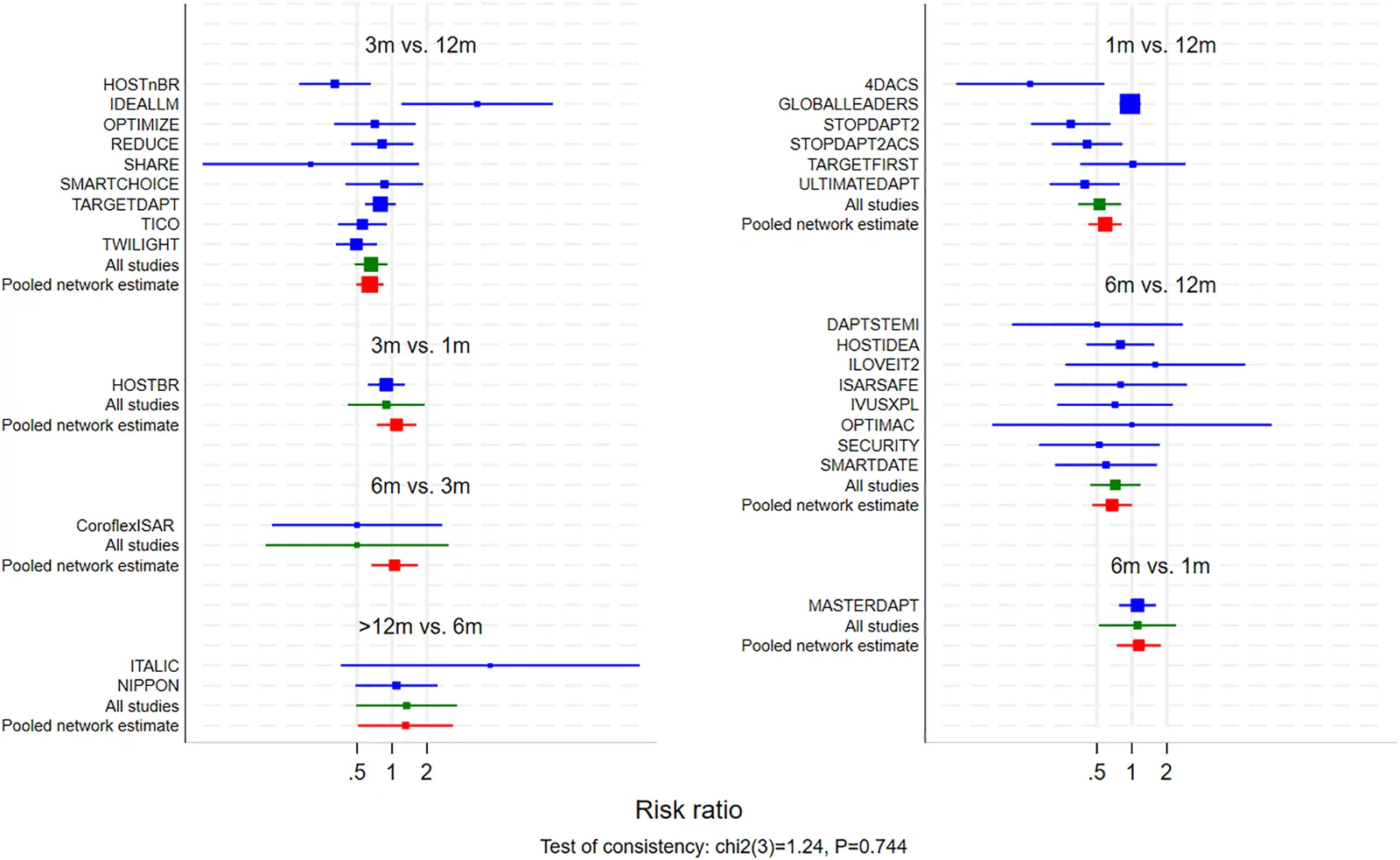
Forest plots comparing dual antiplatelet therapy durations for major bleeding. Forest plots showing pairwise comparisons of dual antiplatelet therapy (DAPT) durations for major bleeding derived from the network meta-analysis. Blue squares represent individual trial estimates with corresponding 95% confidence intervals (horizontal lines). Green squares represent pooled direct estimates from conventional meta-analyses, and red squares represent pooled network estimates. The size of the squares is proportional to study weight. Risk ratios (RRs) are presented on a logarithmic scale, with direction of effect determined by the order of treatment comparison. The vertical reference line indicates no difference (RR = 1). m = months.

Shorter DAPT durations were associated with lower bleeding risk compared with the 12-month reference. Specifically, 1-month DAPT was associated with a significantly reduced risk of bleeding (RR 0.59, 95% CI: 0.42–0.82), as was 3-month DAPT (RR 0.64, 95% CI: 0.49–0.84). The 6-month strategy showed a similar trend (RR 0.68, 95% CI: 0.46–1.00), although this did not reach conventional statistical significance. In contrast, extended DAPT (>12 months) was not associated with a significant difference in bleeding risk (RR 0.89, 95% CI: 0.32–2.45) (Fig 6).

**Fig 7:**
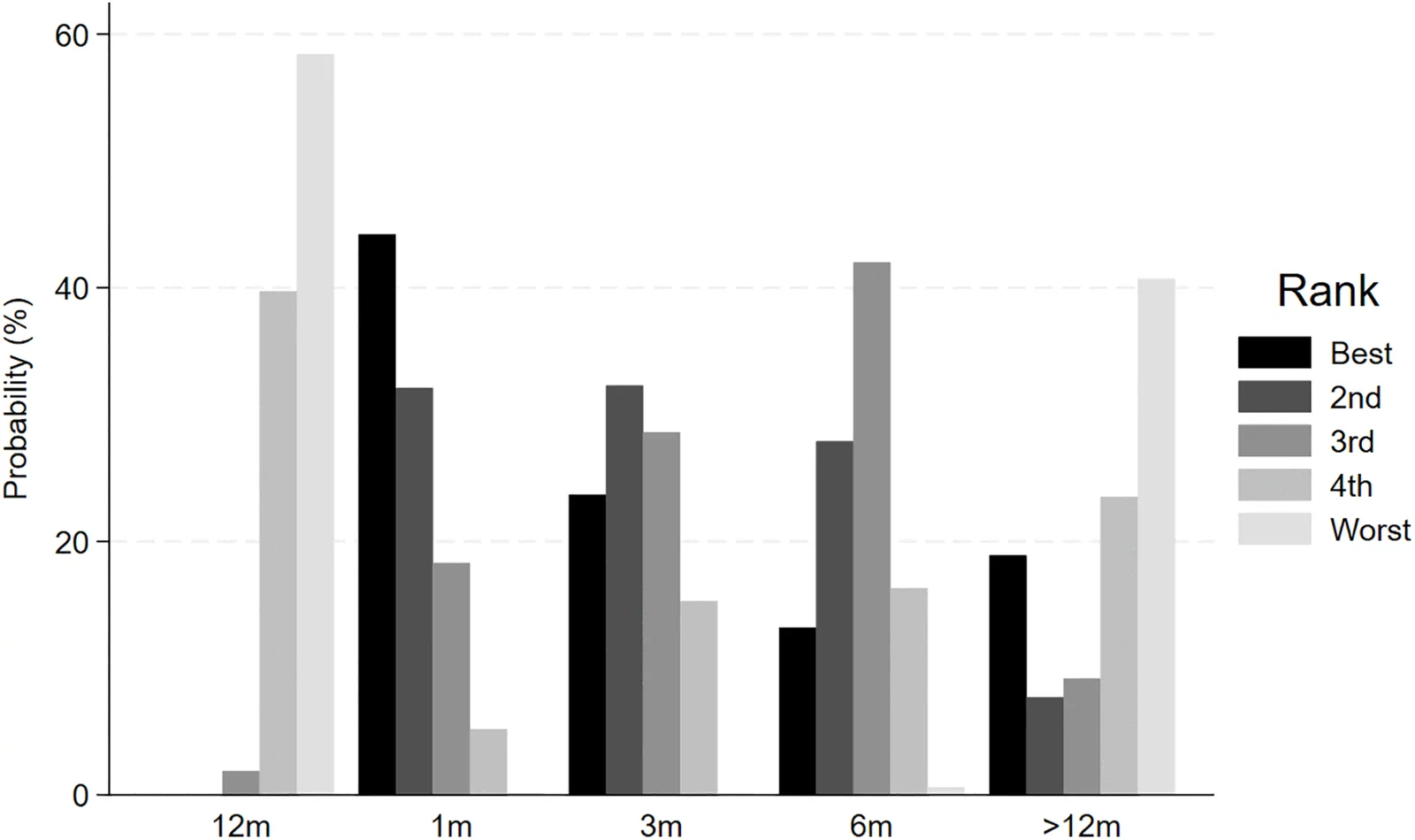
Surface Under the Cumulative Ranking Curve (SUCRA)- based ranking probabilities of dual antiplatelet therapy durations for major bleeding. Bar chart shows the probability of each dual antiplatelet therapy (DAPT) duration strategy ranking at each position (best, second, third, fourth, or worst) for major bleeding, based on the network meta-analysis. Ranking probabilities were derived from the relative treatment effects across the network using a frequentist framework. Each bar represents the probability of a given DAPT duration (1 month, 3 months, 6 months, 12 months, and >12 months) being ranked at a specific position. The “best” rank indicates the lowest risk of major bleeding, whereas the “worst” rank indicates the highest risk.

SUCRA ranking analysis demonstrated that the 1-month DAPT duration had the highest probability of being the safest regimen (44.2%), followed by the 3-month strategy (23.7%). In contrast, 12-month (58.4%) and >12-month (40.7%) durations were most likely to rank as the least safe strategies (Fig 7).

### Publication bias

Comparison-adjusted funnel plots for both MACE and major bleeding demonstrated symmetric distributions of studies (S1 Fig). Consistently, network-wide adaptations of Egger’s regression test showed no significant evidence of small-study effects for either outcome (MACE: p = 0.63; major bleeding: p = 0.20).

### Certainty of evidence

Using the GRADE framework, the overall certainty of evidence was rated as moderate for the efficacy outcome (MACE) and high for the safety outcome (major bleeding). The certainty rating for MACE was downgraded for inconsistency (borderline global network-wide inconsistency and varying clinical trial definitions of MACE), and imprecision (wide confidence intervals that span unity and fail to rule out clinical harm or benefit).

In contrast, the certainty of evidence for major bleeding was rated as high, reflecting the consistency of findings across the network and the robustness of treatment effects.

## Discussion

In this network meta-analysis of 28 RCTs encompassing 84,325 patients, we found that abbreviated DAPT durations (1 and 3 months) following PCI with contemporary DES were associated with a lower risk of major bleeding risk without evidence of increased ischemic risk compared to 12 months. While a 12-month DAPT regimen is the conventional standard of care [12], our findings suggest that shorter durations offer a more favorable safety profile, with no statistically significant difference in MACE across all studied timeframes.

The comparable ischemic protection observed between abbreviated and conventional DAPT durations likely stems from the iterative evolution of stent technology [1]. Contemporary thin-strut DES, characterized by biocompatible fluoropolymers and optimized drug-release kinetics, promote faster and more complete endothelialization. This technological maturation, coupled with more potent P2Y12 inhibitors, has effectively shifted the risk-benefit ratio toward minimizing systemic antithrombotic exposure [1–3].

SUCRA rankings indicated that the 3-month strategy had the highest probability of favorable ranking for MACE. The consistent direction of this finding across the primary and sensitivity analyses suggests that the 3-month duration may represent a clinically relevant threshold at which ischemic protection is preserved while bleeding risk is reduced. However, these findings reflect largely overlapping effect estimates and should be viewed as hypothesis-generating rather than evidence of superiority. Furthermore, the initial network inconsistency identified in the 3-month node was successfully resolved through stratification by study setting (single-country vs. multi-country trials). This suggests that regional practice patterns, such as the East Asian Paradox where patients exhibit higher bleeding but lower ischemic risks [51], or specific patient selection criteria in localized trials may influence outcomes more than DAPT duration alone.

Conversely, safety outcomes demonstrated a clear biological gradient, with progressively shorter DAPT durations associated with lower risk of major bleeding. The 1-month and 3-month strategies emerged as the most favorable for minimizing major bleeding, which is critical given that post-PCI bleeding is a powerful independent predictor of long-term mortality [1–3].

Compared with prior studies and current guidelines, our results extend the findings of individual landmark trials by providing a high-powered, simultaneous comparison of multiple durations. While individual trials were often underpowered to detect differences in infrequent endpoints and frequently relied on composite measures such as net clinical adverse events, our network meta-analysis pools data across studies and separately evaluates ischemic and bleeding outcomes across commonly used DAPT durations, thereby providing more precise estimates and greater granularity in understanding the trade-offs between these competing risks. These findings align with the latest ACC/AHA and ESC guidelines, which increasingly advocate for individualized DAPT durations [12,13], while offering additional comparative insight to inform the selection among abbreviated strategies.

From a clinical perspective, our study findings support the use of shorter DAPT durations in appropriately selected patients, particularly those at elevated bleeding risk. The absence of a meaningful increase in ischemic events with abbreviated DAPT reinforces the safety of shorter regimens in contemporary practice. In this context, early discontinuation of aspirin with continuation of high potent P2Y12 inhibitor monotherapy represents a reasonable treatment approach supported by current evidence [52,53].

Importantly, our analysis extends beyond current guidelines by providing a comparative insight across commonly used abbreviated DAPT strategies; we demonstrate no clear ischemic advantage of one specific regimen over another, while highlighting a consistent pattern of reduced bleeding with shorter courses. Rather than identifying a single optimal duration, this metanalysis underscores that different abbreviated strategies may be tailored to individual patient characteristics. In alignment with current recommendations, shorter regimens may be favored in patients with higher bleeding risk, whereas longer courses remain appropriate for those with elevated ischemic risk or complex coronary anatomy [12,13]. Consistent with this individualized approach, the recently published DAPT-MVD trial demonstrated an ischemic benefit of extending DAPT beyond 12 months in selected patients with multivessel coronary disease [54].

Overall, this study findings supports a transition from fixed-duration DAPT toward a more flexible, patient-centered model, where treatment decisions are guided by individualized risk assessment rather than convention alone.

## Strenghts and limitations

The primary strength of this study lies in its scale and the use of network meta-analysis to generate indirect comparisons where head-to-head trials are lacking. Also, by focusing strictly on trials utilizing contemporary DES, we ensured the findings are applicable to modern interventional practice.

However, several limitations persist. Our use of study-level data precludes the granular analysis of patient-level confounders, such as complexity of coronary anatomy or specific P2Y12 inhibitor selection. Furthermore, post-DAPT antiplatelet strategies varied across trials, with patients transitioning to aspirin, P2Y12 inhibitor monotherapy, or protocol-specific regimens, representing an additional source of heterogeneity that could not be accounted for in this analysis. Additionally, the heterogeneity in outcome definitions across trials and the reliance on subgroup analysis to resolve efficacy inconsistency mean that residual confounding cannot be entirely excluded.

## Conclusion

In patients undergoing PCI with contemporary DES, shorter DAPT durations (1- & 3-month) were associated with reduced bleeding without evidence of increased ischemic risk compared with the conventional 12-month strategy. Among the abbreviated strategies, the 3-month regimen consistently demonstrated a more favorable overall profile across analyses, suggesting it may represent a pragmatic threshold for DAPT abbreviation; however, these findings should be interpreted cautiously given the absence of statistically significant differences in efficacy and the limitations inherent to study-level network meta-analysis. Overall, these results provide comparative evidence across abbreviated DAPT strategies, offering additional insight to refine selection among commonly used regimens in contemporary practice, while reinforcing that DAPT duration should remain individualized based on clinical presentation, ischemic and bleeding risk, procedural complexity, and the planned post-DAPT antiplatelet strategy.

## Supporting information

S1 Fig Comparison-adjusted funnel plot for assessment of small-study effects in the efficacy (MACE) and safety (major bleeding) networks. Visual inspection of the comparison-adjusted funnel plots demonstrated symmetric distributions for both outcomes, MACE (Panel A) and major bleeding (Panel B), with no evidence of small-study effects, consistent with Egger’s regression test (MACE: p = 0.63; major bleeding: p = 0.20). A: 12-month, B: 1-month, C: 3-month, D: 6-month, E: > 12-month.
(TIFF)

S2 Fig Ranking probabilities of DAPT duration strategies across primary and sensitivity analyses. Panel A: Sensitivity analysis restricted to trials enrolling exclusively patients with acute coronary syndrome (ACS). Panel B: Sensitivity analysis restricted to trials not exclusively enrolling ACS patients (<100% ACS; mixed and stable-dominant populations). Panel C: Sensitivity analysis restricted to trials reporting standardized hard ischemic endpoints (death, myocardial infarction, and stroke). Panel D: Primary analysis including trials reporting composite MACE (soft ischemic endpoints). Bar plots display the probability of each DAPT duration (1-month, 3-month, 6-month, and 12-month) being ranked best, second, third, or worst based on network meta-analysis. Higher probabilities of being ranked “best” indicate greater likelihood of optimal performance for reducing MACE.
(TIFF)

S1 Table Detailed trial characteristics, outcome definitions, and bleeding criteria of included randomized trials. Detailed characteristics of randomized controlled trials included in the network meta-analysis, presented by treatment arm. For each study, information is provided on dual antiplatelet therapy (DAPT) duration, antiplatelet regimen during DAPT, post-DAPT single antiplatelet therapy (SAPT), sample size, and baseline patient characteristics. Baseline variables include mean/median age, female proportion, prevalence of diabetes mellitus, proportion of patients presenting with acute coronary syndrome (ACS), and high bleeding risk status (as defined in the original trials). Definitions of major adverse cardiovascular events (MACE) and bleeding criteria are reported as specified in each trial. For the HOST-BR trial, high bleeding risk (^#^) and non-high bleeding (^£^) risk populations are presented separately and treated as independent comparisons. ACS = acute coronary syndrome; asa = aspirin; BARC = Bleeding Academic Research Consortium; CABG = coronary artery bypass grafting; clop = clopidogrel; DAPT = dual antiplatelet therapy; GUSTO = Global Use of Strategies to Open Occluded Coronary Arteries; MACE = major adverse cardiovascular events; MI = myocardial infarction; na = not applicable; p2y12=P2Y12 inhibitor; pras = prasugrel; SAPT = single antiplatelet therapy; ST = stent thrombosis; TIMI = Thrombolysis in Myocardial Infarction; TLR = target lesion revascularization; TVR = target vessel revascularization; tica = ticagrelor.
(DOCX)

S2 Table Full node-splitting results. (DOCX)

S3 Table Sensitivity analyses for MACE across clinical subgroups and endpoint definitions. Sensitivity analyses evaluating the association between DAPT duration and major adverse cardiovascular events (MACE) across clinical subgroups and endpoint definitions. Risk ratios (RR) are presented relative to the 12-month DAPT strategy.
(DOCX)

S1 Checklist PRISMA 2020 checklist. Completed PRISMA 2020 checklist for reporting of this systematic review and network meta-analysis.
(DOCX)

## References

[1] Palmerini T, Biondi-Zoccai G, Della Riva D, Mariani A, Genereux P, Branzi A, et al. Stent thrombosis with drug-eluting stents: is the paradigm shifting?. J Am Coll Cardiol. 2013;62(21):1915–21. doi: 10.1016/j.jacc.2013.08.725 24036025

[2] Rodriguez F, Harrington RA. Management of antithrombotic therapy after acute coronary syndromes. N Engl J Med. 2021;384(5):452–60. doi: 10.1056/NEJMra1607714 33534976 PMC9275395

[3] Marlevi D, Edelman ER. Vascular lesion–specific drug delivery systems: JACC state-of-the-art review. J Am Coll Cardiol 2021;77:2413–31. doi: 10.1016/j.jacc.2021.03.307 33985687 PMC8238531

[4] Ge Z, Kan J, Gao X, Raza A, Zhang J-J, Mohydin BS, et al. Ticagrelor alone versus ticagrelor plus aspirin from month 1 to month 12 after percutaneous coronary intervention in patients with acute coronary syndromes (ULTIMATE-DAPT): a randomised, placebo-controlled, double-blind clinical trial. Lancet. 2024;403(10439):1866–78. doi: 10.1016/S0140-6736(24)00473-2 38599220

[5] Watanabe H, Domei T, Morimoto T, Natsuaki M, Shiomi H, Toyota T, et al. Effect of 1-month dual antiplatelet therapy followed by clopidogrel vs 12-month dual antiplatelet therapy on cardiovascular and bleeding events in patients receiving PCI: the STOPDAPT-2 randomized clinical trial. JAMA. 2019;321(24):2414–27. doi: 10.1001/jama.2019.8145 31237644 PMC6593641

[6] Watanabe H, Morimoto T, Natsuaki M, Yamamoto K, Obayashi Y, Ogita M, et al. Comparison of clopidogrel monotherapy after 1 to 2 months of dual antiplatelet therapy with 12 months of dual antiplatelet therapy in patients with acute coronary syndrome: the STOPDAPT-2 ACS randomized clinical trial. JAMA Cardiol. 2022;7(4):407–17. doi: 10.1001/jamacardio.2021.5244 35234821 PMC8892373

[7] Tarantini G, Honton B, Paradies V, Lemesle G, Range G, Godin M, et al. Early discontinuation of aspirin after PCI in low-risk acute myocardial infarction. N Engl J Med. 2025;393(21):2083–94. doi: 10.1056/NEJMoa2508808 40888726

[8] Hahn J-Y, Song YB, Oh J-H, Chun WJ, Park YH, Jang WJ, et al. Effect of P2Y12 inhibitor monotherapy vs dual antiplatelet therapy on cardiovascular events in patients undergoing percutaneous coronary intervention: the SMART-CHOICE randomized clinical trial. JAMA. 2019;321(24):2428–37. doi: 10.1001/jama.2019.8146 31237645 PMC6593635

[9] Mehran R, Baber U, Sharma SK, Cohen DJ, Angiolillo DJ, Briguori C, et al. Ticagrelor with or without aspirin in high-risk patients after PCI. N Engl J Med. 2019;381(21):2032–42. doi: 10.1056/NEJMoa1908419 31556978

[10] Kim B-K, Hong S-J, Cho Y-H, Yun KH, Kim YH, Suh Y, et al. Effect of ticagrelor monotherapy vs ticagrelor with aspirin on major bleeding and cardiovascular events in patients with acute coronary syndrome: the TICO randomized clinical trial. JAMA. 2020;323(23):2407–16. doi: 10.1001/jama.2020.7580 32543684 PMC7298605

[11] Kang J, Park KW, Han J-K, Hwang D, Yang H-M, Park S, et al. Dual antiplatelet therapy after percutaneous coronary intervention according to bleeding risk (HOST-BR): an open-label, multicentre, randomised clinical trial. Lancet. 2025;406(10516):2244–56. doi: 10.1016/S0140-6736(25)01571-5 41631724

[12] Rao SV, O’Donoghue ML, Ruel M, Rab T, Tamis-Holland JE, Alexander JH, et al. 2025 ACC/AHA/ACEP/NAEMSP/SCAI guideline for the management of patients with acute coronary syndromes: a report of the american college of cardiology/american heart association joint committee on clinical practice guidelines. Circulation. 2025;151(13):e771–862. doi: 10.1161/CIR.0000000000001309 40014670

[13] Valgimigli M, Bueno H, Byrne RA, Collet J-P, Costa F, Jeppsson A, et al. 2017 ESC focused update on dual antiplatelet therapy in coronary artery disease developed in collaboration with EACTS: The Task Force for dual antiplatelet therapy in coronary artery disease of the European Society of Cardiology (ESC) and of the European Association for Cardio-Thoracic Surgery (EACTS). Eur Heart J. 2018;39(3):213–60. doi: 10.1093/eurheartj/ehx419 28886622

[14] PROSPERO. 2023. Accessed 2026 April 4. https://www.crd.york.ac.uk/PROSPERO/view/CRD420261349664

[15] Page MJ, McKenzie JE, Bossuyt PM, Boutron I, Hoffmann TC, Mulrow CD, et al. The PRISMA 2020 statement: an updated guideline for reporting systematic reviews. BMJ. 2021;372:n71. doi: 10.1136/bmj.n71 33782057 PMC8005924

[16] Yang H, Zhang F, Zhang X, Wu Y, Zhong Z, Wang J, et al. Three vs 12-month DAPT after implantation of biodegradable-polymer sirolimus-eluting coronary stent: a randomised clinical trial. BMC Med. 2025;23(1):690. doi: 10.1186/s12916-025-04515-y 41444578 PMC12729390

[17] Hahn J-Y, Song YB, Oh J-H, Cho D-K, Lee JB, Doh J-H, et al. 6-month versus 12-month or longer dual antiplatelet therapy after percutaneous coronary intervention in patients with acute coronary syndrome (SMART-DATE): a randomised, open-label, non-inferiority trial. Lancet. 2018;391(10127):1274–84. doi: 10.1016/S0140-6736(18)30493-8 29544699

[18] Min P-K, Kang TS, Cho Y-H, Cheong S-S, Kim B-K, Kwon SW, et al. P2Y12 inhibitor monotherapy vs dual antiplatelet therapy after deployment of a drug-eluting stent: the SHARE randomized clinical trial. JAMA Netw Open. 2024;7(3):e240877. doi: 10.1001/jamanetworkopen.2024.0877 38451525 PMC10921250

[19] Colombo A, Chieffo A, Frasheri A, Garbo R, Masotti-Centol M, Salvatella N. Second-generation drug-eluting stent implantation followed by 6- versus 12-month dual antiplatelet therapy. J Am Coll Cardiol. 2014;64:2086–97. doi: 10.1016/j.jacc.2014.09.008 25236346

[20] De Luca G, Damen SA, Camaro C, Benit E, Verdoia M, Rasoul S, et al. Final results of the randomised evaluation of short-term dual antiplatelet therapy in patients with acute coronary syndrome treated with a new-generation stent (REDUCE trial). EuroIntervention. 2019;15(11):e990–8. doi: 10.4244/EIJ-D-19-00539 31422929

[21] Feres F, Costa RA, Abizaid A, Leon MB, Marin-Neto JA, Botelho RV, et al. Three vs twelve months of dual antiplatelet therapy after zotarolimus-eluting stents: the OPTIMIZE randomized trial. JAMA. 2013;310(23):2510–22. doi: 10.1001/jama.2013.282183 24177257

[22] Lee B-K, Kim J-S, Lee O-H, Min P-K, Yoon Y-W, Hong B-K, et al. Safety of six-month dual antiplatelet therapy after second-generation drug-eluting stent implantation: OPTIMA-C Randomised Clinical Trial and OCT Substudy. EuroIntervention. 2018;13(16):1923–30. doi: 10.4244/EIJ-D-17-00792 29104179

[23] Hong SJ, Kim JS, Hong SJ, Lim DS, Lee SY, Yun KH, et al. 1-Month dual-antiplatelet therapy followed by aspirin monotherapy after polymer-free drug-coated stent implantation: one-month DAPT trial. JACC Cardiovasc Interv 2021;14:1801–11. doi: 10.1016/j.jcin.2021.06.003 34332946

[24] Nakamura M, Iijima R, Ako J, Shinke T, Okada H, Ito Y. Dual antiplatelet therapy for 6 versus 18 months after biodegradable polymer drug-eluting stent implantation. JACC Cardiovasc Interv. 2017;10:1189–98. doi: 10.1016/j.jcin.2017.04.019 28641838

[25] Valgimigli M, Frigoli E, Heg D, Tijssen J, Jüni P, Vranckx P, et al. Dual antiplatelet therapy after PCI in patients at high bleeding risk. N Engl J Med. 2021;385(18):1643–55. doi: 10.1056/NEJMoa2108749 34449185

[26] Hong SJ, Shin DH, Kim JS, Kim BK, Ko YG, Choi D, et al. 6-Month versus 12-month dual-antiplatelet therapy following long everolimus-eluting stent implantation: the IVUS-XPL randomized clinical trial. JACC Cardiovasc Interv. 2016;9:1438–46. doi: 10.1016/j.jcin.2016.04.036 27212028

[27] Gilard M, Barragan P, Noryani AAL, Noor HA, Majwal T, Hovasse T, et al. 6- versus 24-month dual antiplatelet therapy after implantation of drug-eluting stents in patients nonresistant to aspirin: the randomized, multicenter ITALIC trial. J Am Coll Cardiol. 2015;65(8):777–86. doi: 10.1016/j.jacc.2014.11.008 25461690

[28] Schulz-Schüpke S, Byrne RA, Ten Berg JM, Neumann F-J, Han Y, Adriaenssens T, et al. ISAR-SAFE: a randomized, double-blind, placebo-controlled trial of 6 vs. 12 months of clopidogrel therapy after drug-eluting stenting. Eur Heart J. 2015;36(20):1252–63. doi: 10.1093/eurheartj/ehu523 25616646

[29] Qi J, Li Y, Li J, Jing Q, Xu K, Gao C, et al. Safety and efficacy of 6-month versus 12-month dual antiplatelet therapy in patients after implantation of multiple biodegradable polymer-coated sirolimus-eluting coronary stents: Insight from the I-LOVE-IT 2 trial. Catheter Cardiovasc Interv. 2017;89(S1):555–64. doi: 10.1002/ccd.26947 28318138

[30] van Geuns R-J, Chun-Chin C, McEntegart MB, Merkulov E, Kretov E, Lesiak M, et al. Bioabsorbable polymer drug-eluting stents with 4-month dual antiplatelet therapy versus durable polymer drug-eluting stents with 12-month dual antiplatelet therapy in patients with left main coronary artery disease: the IDEAL-LM randomised trial. EuroIntervention. 2022;17(18):1467–76. doi: 10.4244/EIJ-D-21-00514 35285803 PMC9900447

[31] Han J-K, Hwang D, Yang S, Park S-H, Kang J, Yang H-M, et al. Comparison of 3- to 6-month versus 12-month dual antiplatelet therapy after coronary intervention using the contemporary drug-eluting stents with ultrathin struts: the HOST-IDEA randomized clinical trial. Circulation. 2023;147(18):1358–68. doi: 10.1161/CIRCULATIONAHA.123.064264 36871230

[32] Vranckx P, Valgimigli M, Jüni P, Hamm C, Steg PG, Heg D, et al. Ticagrelor plus aspirin for 1 month, followed by ticagrelor monotherapy for 23 months vs aspirin plus clopidogrel or ticagrelor for 12 months, followed by aspirin monotherapy for 12 months after implantation of a drug-eluting stent: a multicentre, open-label, randomised superiority trial. Lancet. 2018;392(10151):940–9. doi: 10.1016/S0140-6736(18)31858-0 30166073

[33] Kedhi E, Fabris E, van der Ent M, Buszman P, von Birgelen C, Roolvink V, et al. Six months versus 12 months dual antiplatelet therapy after drug-eluting stent implantation in ST-elevation myocardial infarction (DAPT-STEMI): randomised, multicentre, non-inferiority trial. BMJ. 2018;363:k3793. doi: 10.1136/bmj.k3793 30279197 PMC6167608

[34] Jin U, Seo K-W, Yang H-M, Lim H-S, Choi B-J, Choi S-Y, et al. Efficacy and safety of 3 versus 6 months of dual-antiplatelet therapy in patients implanted with a coroflex ISAR stents: a prospective, multicenter, randomized clinical trial. J Invasive Cardiol. 2022;34(9):E653–9. doi: 10.25270/jic/21.00330 35863061

[35] Jang Y, Park S-D, Lee JP, Choi SH, Kong MG, Won YS, et al. One-month dual antiplatelet therapy followed by prasugrel monotherapy at a reduced dose: the 4D-ACS randomised trial. EuroIntervention. 2025;21(14):e796–809. doi: 10.4244/EIJ-D-25-00331 40392195 PMC12285397

[36] Park S-J, Park D-W, Kim Y-H, Kang S-J, Lee S-W, Lee CW, et al. Duration of dual antiplatelet therapy after implantation of drug-eluting stents. N Engl J Med. 2010;362(15):1374–82. doi: 10.1056/NEJMoa1001266 20231231

[37] Bonaca MP, Bhatt DL, Cohen M, Steg PG, Storey RF, Jensen EC, et al. Long-term use of ticagrelor in patients with prior myocardial infarction. N Engl J Med. 2015;372(19):1791–800. doi: 10.1056/NEJMoa1500857 25773268

[38] Lee CW, Ahn J-M, Park D-W, Kang S-J, Lee S-W, Kim Y-H, et al. Optimal duration of dual antiplatelet therapy after drug-eluting stent implantation: a randomized, controlled trial. Circulation. 2014;129(3):304–12. doi: 10.1161/CIRCULATIONAHA.113.003303 24097439

[39] Helft G, Steg PG, Le Feuvre C, Georges J-L, Carrie D, Dreyfus X, et al. Stopping or continuing clopidogrel 12 months after drug-eluting stent placement: the OPTIDUAL randomized trial. Eur Heart J. 2016;37(4):365–74. doi: 10.1093/eurheartj/ehv481 26364288

[40] Li Y, Li J, Wang B, Jing Q, Zeng Y, Hou A, et al. Extended clopidogrel monotherapy vs DAPT in patients with acute coronary syndromes at high ischemic and bleeding risk: the OPT-BIRISK randomized clinical trial. JAMA Cardiol. 2024;9(6):523–31. doi: 10.1001/jamacardio.2024.0534 38630489 PMC11024736

[41] Mauri L, Kereiakes DJ, Yeh RW, Driscoll-Shempp P, Cutlip DE, Steg PG, et al. Twelve or 30 months of dual antiplatelet therapy after drug-eluting stents. N Engl J Med. 2014;371(23):2155–66. doi: 10.1056/NEJMoa1409312 25399658 PMC4481318

[42] Kereiakes DJ, Yeh RW, Massaro JM, Driscoll-Shempp P, Cutlip DE, Steg PG, et al. Antiplatelet therapy duration following bare metal or drug-eluting coronary stents: the dual antiplatelet therapy randomized clinical trial. JAMA. 2015;313(11):1113–21. doi: 10.1001/jama.2015.1671 25781440 PMC4481320

[43] Collet J-P, Silvain J, Barthélémy O, Rangé G, Cayla G, Van Belle E, et al. Dual-antiplatelet treatment beyond 1 year after drug-eluting stent implantation (ARCTIC-Interruption): a randomised trial. Lancet. 2014;384(9954):1577–85. doi: 10.1016/S0140-6736(14)60612-7 25037988

[44] Hong S-J, Lee S-J, Suh Y, Yun KH, Kang TS, Shin S, et al. Stopping aspirin within 1 month after stenting for ticagrelor monotherapy in acute coronary syndrome: the T-PASS randomized noninferiority trial. Circulation. 2024;149(8):562–73. doi: 10.1161/CIRCULATIONAHA.123.066943 37878786

[45] Natsuaki M, Watanabe H, Morimoto T, Yamamoto K, Obayashi Y, Nishikawa R, et al. An aspirin-free versus dual antiplatelet strategy for coronary stenting: STOPDAPT-3 randomized trial. Circulation. 2024;149(8):585–600. doi: 10.1161/CIRCULATIONAHA.123.066720 37994553

[46] Guimarães PO, Franken M, Tavares CAM, Antunes MO, Silveira FS, Andrade PB, et al. Early withdrawal of aspirin after PCI in acute coronary syndromes. N Engl J Med. 2025;393(21):2095–106. doi: 10.1056/NEJMoa2507980 40888723

[47] Natsuaki M, Morimoto T, Yamamoto E, Shiomi H, Furukawa Y, Abe M, et al. One-year outcome of a prospective trial stopping dual antiplatelet therapy at 3 months after everolimus-eluting cobalt-chromium stent implantation: ShortT and OPtimal duration of Dual AntiPlatelet Therapy after everolimus-eluting cobalt-chromium stent. Cardiovasc Interv Ther 2016;31:196–209. doi: 10.1007/S12928-015-0366-9 26518420 PMC4923071

[48] Valgimigli M, Campo G, Monti M, Vranckx P, Percoco G, Tumscitz C, et al. Short- versus long-term duration of dual-antiplatelet therapy after coronary stenting: a randomized multicenter trial. Circulation. 2012;125(16):2015–26. doi: 10.1161/CIRCULATIONAHA.111.071589 22438530

[49] Kim B-K, Hong M-K, Shin D-H, Nam C-M, Kim J-S, Ko Y-G, et al. A new strategy for discontinuation of dual antiplatelet therapy: the RESET Trial (REal Safety and Efficacy of 3-month dual antiplatelet Therapy following Endeavor zotarolimus-eluting stent implantation). J Am Coll Cardiol. 2012;60(15):1340–8. doi: 10.1016/j.jacc.2012.06.043 22999717

[50] Gwon H-C, Hahn J-Y, Park KW, Song YB, Chae I-H, Lim D-S, et al. Six-month versus 12-month dual antiplatelet therapy after implantation of drug-eluting stents: the Efficacy of Xience/Promus Versus Cypher to Reduce Late Loss After Stenting (EXCELLENT) randomized, multicenter study. Circulation. 2012;125(3):505–13. doi: 10.1161/CIRCULATIONAHA.111.059022 22179532

[51] Gong Y, Jeong YH, Wang TD, Tan JWC, Qian J, Yan H, et al. Position statement on antiplatelet therapy for East Asians with coronary artery disease: 2025 update. JACC: Asia. 2025;5:821–46. doi: 10.1016/j.jacasi.2025.04.010 40610120 PMC12277199

[52] Galli M, Gragnano F, Berteotti M, Marcucci R, Gargiulo G, Calabrò P, et al. Antithrombotic therapy in high bleeding risk, part i: percutaneous cardiac interventions. JACC Cardiovasc Interv. 2024;17(19):2197–215. doi: 10.1016/j.jcin.2024.08.022 39415380

[53] Galli M, Laudani C, Occhipinti G, Spagnolo M, Gragnano F, D’Amario D, et al. P2Y12 inhibitor monotherapy after short DAPT in acute coronary syndrome: a systematic review and meta-analysis. Eur Heart J Cardiovasc Pharmacother. 2024;10(7):588–98. doi: 10.1093/ehjcvp/pvae057 39054275

[54] Tian J, Wang Z, Wang Y, Wang F, Peng X, Xiao J, et al. Extended dual antiplatelet therapy for multivessel coronary artery disease. N Engl J Med. 2026;395(3):233–42. doi: 10.1056/NEJMoa2517588 42456136

