# Supplementary material for: Dual antiplatelet therapy duration after percutaneous coronary intervention with contemporary drug-eluting stents: A systematic review and network meta-analysis of randomized trials
Source: PLoS One. 2026 Sep 2;21(9):e0357462. doi: 10.1371/journal.pone.0357462 (PMC13537542; doi:10.1371/journal.pone.0357462)
Supplement: Supplementary file 1 [file pone.0357462.s003.docx]

Supplementary Table S1. **Detailed trial characteristics, outcome definitions, and bleeding criteria of included randomized trials.**

| **Trial** | **DAPT duration (months)** | **DAPT used** | **SAPT after DAPT** | **Patients included.** | **Mean Age** | **Female proportion** | **Diabetes proportion** | **ACS proportion** | **High bleeding risk** | **MACE definition** | **Bleeding Criteria** |
| --- | --- | --- | --- | --- | --- | --- | --- | --- | --- | --- | --- |
| TICO; Byeong-Keuk Kim; JAMA 2020 | 3 | tica+asa | tica | 1527 | 61 | 21 | 27 | 100 | mixed | (Death/ MI/ ST/ stroke/ TVR) | TIMI major |
|  | 12 | tica+asa | na | 1529 | 61 | 20 | 27 | 100 | mixed |  |  |
| REDUCE; Giuseppe De Luca; EuroInterventions 2019 | 3 | pras/tica+asa | asa/p2y12 | 751 | 61 | 17.4 | 21.6 | 100 | mixed | (All-cause mortality/ definitive or probable ST/ stroke/ MI/ TVR) | BARC 2/3/5 |
|  | 12 | pras/tica+asa | na | 745 | 60 | 22.7 | 19.5 | 100 | mixed |  |  |
| DAPT-STEMI; Elvin Kedhi; BMJ 2018 | 6 | p2y12+asa | asa | 433 | 59.8 | 22 | 13 | 100 | mixed | (Cardiac death/ TVR/ target lesion MI) | BARC 3 |
|  | 12 | p2y12+asa | na | 437 | 60.2 | 24 | 14 | 100 | mixed |  |  |
| ULTIMATE-DAPT; Zhen Ge; Lancet 2024 | 1 | tica+asa | tica | 1700 | 62 | 25.7 | 31.8 | 100 | mixed | (Cardiac death/ MI/ ischemic stroke/ definite ST/ TVR) | BARC 3/5 |
|  | 12 | tica+asa | na | 1700 | 63 | 26.1 | 31.5 | 100 | mixed |  |  |
| STOPDAPT-2; Hirotoshi Watanabe; JAMA 2019 | 1 | clop+asa | clop | 1500 | 68.1 | 21.1 | 39 | 37.7 | mixed | (Cardiovascular death/ MI/ definite ST/ ischemic or hemorrhagic stroke) | BARC 3/5 |
|  | 12 | clop+asa | na | 1509 | 69.1 | 23.5 | 38 | 38.6 | mixed |  |  |
| HOST-BR^#^; Jeehoon Kang; Lancet 2025 | 1 | p2y12+asa | asa/p2y12 | 798 | 76 | 33.2 | 55.1 | 57.9 | yes | (Cardiovascular death/ MI/ definite or probable ST/ ischemic stroke) | BARC 3/5 |
|  | 3 | p2y12+asa | na | 800 | 76 | 33.8 | 51.1 | 58.1 | yes |  |  |
| HOST-BR^£^; Jeehoon Kang; Lancet 2025 | 3 | p2y12+asa | asa/p2y12 | 1649 | 64 | 21 | 32.2 | 62.1 | no | (Cardiovascular death/ MI/ definite or probable ST/ ischemic stroke) | BARC 3/5 |
|  | 12 | p2y12+asa | na | 1650 | 64 | 20.7 | 32.4 | 63.5 | no |  |  |
| STOPDAPT-2 ACS; Hirotoshi Watanabe; JAMA Cardiology 2022 | 1 | pras/clop+asa | clop | 2058 | 67 | 20.8 | 29.5 | 100 | mixed | (Cardiovascular death/ MI/ definite ST/ ischemic or hemorrhagic stroke) | BARC 3/5 |
|  | 12 | pras/clop+asa | na | 2078 | 66.6 | 20.6 | 29.9 | 100 | mixed |  |  |
| TWILIGHT; R. Mehran; NEJM 2019 | 3 | tica/asa | tica | 3555 | 65.2 | 23.8 | 37.1 | 63.9 | yes | (Cardiovascular death/ nonfatal MI/ nonfatal ischemic stroke) | BARC 3/5 |
|  | 12 | tica/asa |  | 3564 | 65.1 | 23.9 | 36.5 | 65.7 | yes |  |  |
| TARGET-FIRST; G. Tarantini; NEJM 2025 | 1 | p2y12+asa | p2y12 | 961 | 61 | 23.2 | 14 | 100 | mixed | (Cardiovascular death/ MI / ischemia-driven TVR) | BARC 3/5 |
|  | 12 | p2y12+asa | na | 981 | 61 | 20.1 | 14.9 | 100 | mixed |  |  |
| SMART-CHOICE; Joo-Yong Hahn; JAMA 2019 | 3 | p2y12+asa | p2y12 | 1495 | 64.6 | 27.3 | 38.2 | 58.2 | mixed | (All-cause mortality/ MI/ stroke) | BARC 3/5 |
|  | 12 | p2y12+asa |  | 1498 | 64.4 | 25.8 | 36.8 | 58.2 | mixed |  |  |
| GLOBAL LEADERS; Pascal Vranckx; Lancet 2018 | 1 | tica+asa | tica | 7980 | 64.5 | 23.4 | 25.7 | 47 | mixed | (All-cause mortality/ stroke/ new Q-wave MI) | BARC 3/5 |
|  | 12 | tica/clop+asa | na | 7988 | 64.6 | 23.1 | 24.9 | 46.8 | mixed |  |  |
| 4D-ACS; Youngwoo Jang; EuroIntervention 2025 | 1 | pras+asa | pras | 328 | 60.4 | 18 | 31.7 | 100 | mixed | (Cardiovascular death/ MI/ stroke/ ischemia-driven TVR) | BARC 3/5 |
|  | 12 | pras+asa | na | 328 | 61.3 | 16.8 | 31.7 | 100 | mixed |  |  |
| HOST-IDEA; Jung-Kyu Han; Circulation 2023 | 6 | p2y12+asa | p2y12/asa | 1002 | 65.6 | 27 | 40.5 | 56.6 | mixed | (All-cause death/ any MI/ any revascularization/ stroke) | BARC 3/5 |
|  | 12 | p2y12+asa |  | 1011 | 65.9 | 25.2 | 37.4 | 53.7 | mixed |  |  |
| MASTER DAPT; M. Valgimigli; NEJM 2021 | 1 | p2y12+asa | p2y12/asa | 2295 | 76.1 | 30.7 | 32.9 | 49.1 | yes | (All-cause death/ MI/ stroke) | BARC 3/5 |
|  | 6 | p2y12+asa |  | 2284 | 76 | 30.8 | 34.3 | 47.4 | yes |  |  |
| SHARE; Pil-Ki Min; JAMA 2024 | 3 | p2y12+asa | tica/clopi | 694 | 62.8 | 22.5 | 32.8 | 73.9 | mixed | (Cardiac death/ MI/ ST/ stroke/ TLR). | BARC 3/5 |
|  | 12 | p2y12+asa | na | 693 | 63.2 | 25.4 | 35.1 | 73.6 | mixed |  |  |
| One-month DAPT; Sung-Jin Hong; JACC: Cardiovascular Interventions 2021 | 1 | clop+asa | asa | 1507 | 67 | 31 | 37 | 38 | mixed | not defined | BARC 3/5 |
|  | 12 | clop+asa | na | 1513 | 67 | 31 | 38 | 41 | mixed |  |  |
| OPTIMA-C; Byoung-Kwon Lee; EuroIntervention 2018 | 6 | clop+asa | asa | 683 | 62.8 | 30 | 29.1 | 50.4 | mixed | (Cardiac death/ TV-related MI/ ischemia-driven TLR) | TIMI major |
|  | 12 | clop+asa | na | 684 | 64.4 | 32.2 | 29.7 | 50.9 | mixed |  |  |
| IVUS-XPL; Sung-Jin Hong; JACC: Cardiovascular Interventions 2016 | 6 | clop+asa | asa | 699 | 63 | 33 | 36 | 49 | mixed | (Cardiac death/ MI/ TLR) | TIMI major |
|  | 12 | clop+asa | na | 701 | 63 | 30 | 37 | 49 | mixed |  |  |
| Coroflex ISAR; Uram Jin; J INVASIVE CARDIOL 2022 | 3 | p2y12+asa | asa | 244 | 64 | 24.2 | 27.5 | 58.2 | mixed | (Cardiovascular death/ non-fatal MI/ TVR) | BARC 2/3/5 |
|  | 6 | p2y12+asa | na | 244 | 64.9 | 27 | 34 | 61.5 | mixed |  |  |
| IDEAL-LM; Robert-Jan van Geuns; EuroIntervention 2022 | 4 | p2y12+asa | asa | 410 | 66.8 | 17.6 | 21.2 | 40.7 | mixed | (All-cause death/ MI/ ischemia-driven TVR). | BARC 3/5 |
|  | 12 | p2y12+asa | na | 408 | 66 | 23.3 | 22.8 | 40.2 | mixed |  |  |
| SMART-DATE; Joo-Yong Hahn; Lancet 2018 | 6 | p2y12+asa | asa | 1357 | 62 | 25.1 | 26.9 | 100 | mixed | (All-cause death/ MI/ stroke) | BARC 3/5 |
|  | 12 | p2y12+asa | na | 1355 | 62.2 | 24.1 | 28.1 | 100 | mixed |  |  |
| ITALIC; Martine Gilard; JACC 2015 | 6 | p2y12+asa | asa | 912 | 61.7 | 19.2 | 36.3 | 58.9 | mixed | not defined | TIMI major |
|  | 24 | p2y12+asa | na | 910 | 61.5 | 20.8 | 37.8 | 58.5 | mixed |  |  |
| NIPPON; Masato Nakamura; JACC: Cardiovascular Interventions 2017 | 6 | p2y12+asa | asa | 1654 | 67.4 | 21.2 | 37.4 | 51.3 | mixed | not defined | BARC 3/5 |
|  | 18 | p2y12+asa | na | 1653 | 67.2 | 20.6 | 38.4 | 55.6 | mixed |  |  |
| SECURITY; Antonio Colombo; JACC 2014 | 6 | p2y12+asa | asa | 682 | 64.9 | 22.4 | 30.2 | 38.4 | mixed | not defined | BARC 3/5 |
|  | 12 | p2y12+asa | na | 717 | 65.5 | 23.2 | 31.1 | 38.4 | mixed |  |  |
| TARGET DAPT; Hongbo Yang; BMC Medicine 2025 | 3 | tica/clop+asa | asa | 1222 | 63.4 | 33.1 | 27.8 | 14.2 | mixed | (All-cause death/ MI/ ischemia-driven TVR) | BARC 2/3/5 |
|  | 12 | tica/clop+asa | na | 1223 | 63.4 | 32.6 | 26.8 | 14.4 | mixed |  |  |
| ISAR-SAFE; Stefanie Schulz-Schu¨pke; European Heart Journal 2015 | 6 | clop+asa | asa | 1997 | 67.2 | 19.3 | 24.8 | 50.7 | mixed | (Death/ MI/ definite or probable ST/ stroke) | TIMI major |
|  | 12 | clop+asa | na | 2003 | 67.2 | 19.5 | 24.2 | 51.6 | mixed |  |  |
| OPTIMIZE; Fausto Feres; JAMA 2013 | 3 | clop+asa | asa | 1563 | 61.3 | 36.5 | 35.4 | 40.2 | mixed | (All-cause death/ MI/ urgent CABG/ TLR) | GUSTO |
|  | 12 | clop+asa | na | 1556 | 61.9 | 36.9 | 35.3 | 41.4 | mixed |  |  |
| I-LOVE-IT 2; Jing Qi; Catheterization and Cardiovascular Interventions 2017 | 6 | clop+asa | asa | 440 | 60.7 | 32.7 | 37 | 80.5 | mixed | (Cardiac death/ TV MI/ clinically indicated TLR) | BARC 3/5 |
|  | 12 | clop+asa | na | 467 | 60.5 | 28.1 | 40.7 | 78.8 | mixed |  |  |

Detailed characteristics of randomized controlled trials included in the network meta-analysis, presented by treatment arm. For each study, information is provided on dual antiplatelet therapy (DAPT) duration, antiplatelet regimen during DAPT, post-DAPT single antiplatelet therapy (SAPT), sample size, and baseline patient characteristics.

Baseline variables include mean/median age, female proportion, prevalence of diabetes mellitus, proportion of patients presenting with acute coronary syndrome (ACS), and high bleeding risk status (as defined in the original trials).

Definitions of major adverse cardiovascular events (MACE) and bleeding criteria are reported as specified in each trial.

For the HOST-BR trial, high bleeding risk (^#^) and non–high bleeding (^£^) risk populations are presented separately and treated as independent comparisons.

ACS=acute coronary syndrome; asa=aspirin; BARC=Bleeding Academic Research Consortium; CABG=coronary artery bypass grafting; clop=clopidogrel; DAPT=dual antiplatelet therapy; GUSTO=Global Use of Strategies to Open Occluded Coronary Arteries; MACE=major adverse cardiovascular events; MI=myocardial infarction; na=not applicable; p2y12=P2Y12 inhibitor; pras=prasugrel; SAPT=single antiplatelet therapy; ST=stent thrombosis; TIMI=Thrombolysis in Myocardial Infarction; TLR=target lesion revascularization; TVR=target vessel revascularization; tica=ticagrelor.
