# Supplementary material for: Dual antiplatelet therapy duration after percutaneous coronary intervention with contemporary drug-eluting stents: A systematic review and network meta-analysis of randomized trials
Source: PLoS One. 2026 Sep 2;21(9):e0357462. doi: 10.1371/journal.pone.0357462 (PMC13537542; doi:10.1371/journal.pone.0357462)
Supplement: Supplementary file 2 [file pone.0357462.s004.docx]

Supplementary Table S2: **Full node-splitting results**

| Side | Direct coefficient | Indirect coefficient | Difference | P-value |
| --- | --- | --- | --- | --- |
| 1- vs. 12- month | -.061 | .248 | -.309 | 0.058 |
| 3- vs. 12- month | .008 | -.524 | .532 | 0.012 |
| 6- vs. 12- month | .068 | .028 | .040 | 0.859 |
| 1- vs. 3- month | -.522 | .065 | -.587 | 0.007 |
| 1- vs. 6- month | .005 | .033 | -.028 | 0.905 |
| 3- vs. 6- month | -6.70e-12 | .111 | -.111 | 0.864 |
