# Supplementary material for: Dual antiplatelet therapy duration after percutaneous coronary intervention with contemporary drug-eluting stents: A systematic review and network meta-analysis of randomized trials
Source: PLoS One. 2026 Sep 2;21(9):e0357462. doi: 10.1371/journal.pone.0357462 (PMC13537542; doi:10.1371/journal.pone.0357462)
Supplement: Supplementary file 3 [file pone.0357462.s005.docx]

Supplementary Table S3. **Sensitivity analyses for MACE across clinical subgroups and endpoint definitions**

| Analysis group | Number of studies | Comparison | RR (95% CI) | p-value |
| --- | --- | --- | --- | --- |
| ACS-only trials | 8 | 1m vs 12m | 1.07 (0.79–1.46) | 0.64 |
|  |  | 3m vs 12m | 0.84 (0.58–1.21) | 0.34 |
|  |  | 6m vs 12m | 1.03 (0.66–1.63) | 0.89 |
|  |  |  |  |  |
| Non-ACS-exclusive trials (<100% ACS) | 16 | 1m vs 12m | 1.02 (0.82–1.27) | 0.83 |
|  |  | 3m vs 12m | 0.97 (0.82–1.15) | 0.76 |
|  |  | 6m vs 12m | 1.06 (0.85–1.32) | 0.59 |
|  |  |  |  |  |
| Composite (soft) MACE trials | 20 | 1m vs 12m | 1.13 (0.90–1.41) | 0.29 |
|  |  | 3m vs 12m | 0.94 (0.81–1.10) | 0.46 |
|  |  | 6m vs 12m | 1.05 (0.83–1.33) | 0.69 |
|  |  |  |  |  |
| Hard ischemic endpoint trials | 4 | 1m vs 12m | 0.89 (0.78–1.02) | 0.09 |
|  |  | 3m vs 12m | 1.17 (0.75–1.81) | 0.47 |
|  |  | 6m vs 12m | 0.96 (0.78–1.19) | 0.70 |

Sensitivity analyses evaluating the association between DAPT duration and major adverse cardiovascular events (MACE) across clinical subgroups and endpoint definitions. Risk ratios (RR) are presented relative to the 12-month DAPT strategy.
