# Supplementary material for: Dual antiplatelet therapy duration after percutaneous coronary intervention with contemporary drug-eluting stents: A systematic review and network meta-analysis of randomized trials
Source: PLoS One. 2026 Sep 2;21(9):e0357462. doi: 10.1371/journal.pone.0357462 (PMC13537542; doi:10.1371/journal.pone.0357462)
Supplement: Supplementary file 5 [file pone.0357462.s008.docx]

Manuscript ID: PONE-D-26-23217

Authors: Hlupeni et al.

**GENERAL RESPONSE**

We thank the Editor and Reviewers for their thoughtful and constructive feedback. We have carefully revised the manuscript to address all comments. All changes have been incorporated into the revised manuscript and are highlighted in the tracked version. Below, we provide a point-by-point response.

**REVIEWER #1**

**Comment 1: Post-DAPT monotherapy strategies vary across trials… stratify or clarify.**

Response: We thank the reviewer for this important observation. We agree that post-DAPT antiplatelet strategies varied across trials and represent a potential source of clinical heterogeneity. Accordingly, we have explicitly acknowledged this heterogeneity in the Methods and Limitations sections, clarifying that treatment groups may include varying post-DAPT monotherapy regimens (aspirin vs. P2Y12 inhibitor).

Given the limited reporting granularity across trials, formal stratification by post-DAPT regimen was not feasible.

**Comment 2: Avoid implying superiority of 3-month DAPT.**

Response: We agree and have revised the manuscript accordingly.

- All language implying superiority has been removed.
- SUCRA findings are now consistently described as probabilistic and exploratory.

**Comment 3: Provide subgroup inconsistency results.**

Response: We have now explicitly reported these results in both the Results section and Supplementary Material: Multicountry trials: p = 0.94, Single-country trials: p = 0.34. Additionally, full node-splitting results have been included in Supplementary Table S2

**Comment 4: GRADE rating too high for MACE.**

Response: We agree and have revised the certainty of evidence. MACE downgraded from High to Moderate. Reasons included inconsistency (borderline global inconsistency) and imprecision (confidence intervals crossing unity)

**Comment 5: Additional sensitivity analyses (ACS vs mixed populations).**

Response: We thank the reviewer for this valuable suggestion. We have now performed and included:

- ACS-only analysis vs Mixed/stable population analysis

Results presented in Main Results section, Supplementary Table S3 and Supplementary Figure S2. Findings were consistent with the primary analysis.

**Comment 6: >12-month node discrepancy between networks.**

Response: We have clarified this in the Results section. The >12-month node was not included in the MACE network because trials evaluating extended DAPT did not report MACE as a standalone endpoint.

**Comment 7: Include recent literature (de-escalation, monotherapy).**

Response: We have expanded the Discussion and added relevant recent studies (PMID: 39054275; PMID: 39415380).

**Comment 8: Conclusion too generic.**

Response: We agree and have revised the conclusion to emphasize:

- Individualization of DAPT duration
- Role of ischemic vs bleeding risk
- Procedural complexity
- Post-DAPT strategy

**Comment 9: Title too long.**

Response: We have shortened the title while preserving key elements.

**REVIEWER #2**

**Comment: Study is not novel and lacks impact.**

Response: We respectfully disagree and have revised the manuscript to better clarify its novelty.

Key contributions of our study:

- Direct comparison across multiple abbreviated DAPT durations (1-, 3-, 6-month). This has not been addressed in prior pairwise meta-analyses
- Separation of ischemic and bleeding outcomes: Prior studies relied heavily on composite endpoints (e.g., NACE)
- Granular comparative framework: Allows clinicians to differentiate between abbreviated strategies

We have strengthened the Introduction and Discussion by including clarifications such as “While current guidelines and prior meta-analyses support DAPT abbreviation, they do not provide direct comparative insight across abbreviated strategies. Our network meta-analysis addresses this gap…”

**REVIEWER #3**

**Major Comment 1: GRADE inconsistency**

Response: Addressed as above, MACE downgraded to Moderate and Node-splitting results added to Supplementary information

**Major Comment 2: MACE heterogeneity threatens transitivity**

Response: We agree and have performed additional sensitivity analysis: Restricted to hard ischemic endpoints (death, MI, stroke). No significant differences observed and findings were consistent with primary analysis, though less precise due to fewer studies in other subgroups. We added these additional analyses to Results section and Supplementary Table S3

**Major Comment 3: Over-reliance on SUCRA**

Response: We agree and revised all relevant sections. We removed language implying “optimal strategy” and emphasized no non-significant differences in MACE outcomes and the fact that SUCRA ranking are probabilistic.

**Major Comment 4: Publication bias not assessed**

Response: We have now included comparison-adjusted funnel plots (Supplementary Figure S1) and Egger’s regression test, MACE: p = 0.63, Bleeding: p = 0.20

**Major Comment 5: Inconsistency resolution not supported**

Response: We have now explicitly reported global inconsistency p-values by subgroup: Multicountry: 0.94 and Single-country: 0.34. Supporting data included in Supplementary materials.

**REVIEWER #4**

**Comment: Clarify novelty and clinical value**

Response: We appreciate this important point and have strengthened the manuscript accordingly.

We now emphasize that our study moves beyond “whether to shorten DAPT” to “how abbreviated strategies compare with one another”. We added clarifications such as “Our analysis provides comparative insight across commonly used abbreviated DAPT strategies, addressing a key gap not resolved by existing guidelines or prior meta-analyses.”
