# Supplementary material for: Dual antiplatelet therapy duration after percutaneous coronary intervention with contemporary drug-eluting stents: A systematic review and network meta-analysis of randomized trials
Source: PLoS One. 2026 Sep 2;21(9):e0357462. doi: 10.1371/journal.pone.0357462 (PMC13537542; doi:10.1371/journal.pone.0357462)
Supplement: Supplementary file 6 [file pone.0357462.s009.docx]

**Manuscript ID:** PONE-D-26-23217R1
**Title:** Dual antiplatelet therapy duration after percutaneous coronary intervention with contemporary drug-eluting stents: A systematic review and network meta-analysis of randomized trials

Dear Dr. Liu and Reviewers,

Thank you for your careful review of our manuscript. We appreciate that Reviewers 1, 3, and 4 considered their previous concerns adequately addressed. We have carefully considered the remaining comments from Reviewer 2 and the Academic Editor and have made additional revisions as detailed below.

**ACADEMIC EDITOR**

**Comment**: *Review 2 pointed out some important trials are not included. Please double check the literature search strategy. Regarding the inclusion of some latest studies, I suggest to do an updated search. They can be included in your discussion if the publication date is later than the deadline of your literature search. The heterogeneity analysis should also be improved.*

**Response**: Thank you for these recommendations. We rechecked our literature search and eligibility assessment and confirmed that the trials specifically identified by Reviewer 2 were captured by our search but excluded according to predefined eligibility criteria. These exclusions were already explicitly reported in the manuscript (Lines 137–140). We also reviewed recently published evidence and have incorporated the DAPT-MVD trial into the Discussion as important contemporary evidence published after our prespecified search period.

Regarding heterogeneity, this concern had been extensively addressed in Revision 1 through global and local inconsistency assessments and additional sensitivity analyses according to ACS presentation and MACE endpoint definition, including an analysis restricted to hard ischemic endpoints. Complete node-splitting results were also provided in the Supporting Information, and the certainty of evidence for MACE was downgraded to moderate because of inconsistency and imprecision. These revisions were considered satisfactory by Reviewers 1, 3, and 4, with Reviewer 3 specifically concluding that the manuscript was methodologically sound. Accordingly, no additional heterogeneity analyses were performed in the present revision.

**REVIEWER #1**

**Comment**: *The manuscript has been substantially revised in accordance with the Reviewers' requests. I have no further comments for the authors.*

**Response:** We thank the Reviewer for the careful review and positive assessment of our revised manuscript.

**REVIEWER #2**

**Comment**: *I continue to see poor novelty. The topic is overbooked and there are hundreds of publications including trials, meta-analyses, expert reviews, consensus documents and international guidelines on the “optimal DAPT”.*

**Response:** We appreciate the Reviewer’s concern and agree that the broad question of optimal DAPT duration has been extensively studied. However, the present study does not seek to re-examine the broad question of “**optimal DAPT duration”** across abbreviated, conventional, and extended treatment strategies. Rather, our research question is specifically focused on DAPT abbreviation (<12 months): how the commonly used 1-, 3-, and 6-month abbreviated regimens compare with one another and whether the available evidence identifies a clinically meaningful threshold for abbreviation in the contemporary DES era. This distinction is important because, although abbreviated DAPT is increasingly supported by randomized trials and current guidelines, the individual abbreviated strategies have predominantly been evaluated against **conventional-duration therapy rather than against one another**. By integrating direct and indirect evidence, our network meta-analysis provides simultaneous comparison of these abbreviated strategies while separately evaluating ischemic and bleeding outcomes. Our prior revision had further emphasized this specific research question in the Introduction and Discussion to avoid conflating DAPT abbreviation with the broader literature on optimal DAPT duration.

**Comment**: *The review is not properly updated given that few weeks ago the DAPT MVD has been published on the NEJM.*

**Response:** We thank the Reviewer for highlighting this important recent trial. DAPT-MVD was published after our prespecified literature-search period and has now been incorporated into the Discussion. DAPT-MVD evaluates extended DAPT beyond 12 months in selected patients with multivessel coronary disease and therefore addresses a **different therapeutic question from the principal focus of our study**, which is the comparative performance and threshold of abbreviated (<12-month) DAPT strategies.

**Comment**: *I also have concerns in terms of studies included, for example I see EXCELLENT, RESET, PRODIGY and DAPT trials missing...*

**Response:** We appreciate the opportunity to clarify this point. These trials were not missed by our literature search; they were identified, evaluated, and excluded according to our predefined eligibility criteria. These exclusions were already explicitly reported in the manuscript (Lines 137-140). Specifically, PRODIGY, RESET, and EXCELLENT are cited as references 48–50, respectively, and were excluded because of **substantial first-generation stent use**, inconsistent with our prespecified contemporary-DES eligibility criterion. The DAPT trial evaluated **extended therapy without an abbreviated (<12-month) DAPT arm** and therefore did not meet the eligibility criteria for the principal research question. We rechecked the literature search and confirmed these exclusions.

**Comment**: *I continue to see impossibility to properly manage the huge heterogeneity across trials designs, definitions, etc. and therefore absence of sufficient quality and novelty to advance our current knowledge.*

**Response**: We agree that heterogeneity across DAPT trials is an important consideration. This concern was extensively addressed in Revision 1 through global and local inconsistency testing and additional sensitivity analyses according to ACS presentation and MACE endpoint definition, including an analysis restricted to standardized hard ischemic endpoints (death, myocardial infarction, and stroke). Complete node-splitting results were provided in the Supporting Information, and the certainty of evidence for MACE was downgraded to moderate because of inconsistency and imprecision. These methodological revisions were considered satisfactory by Reviewers 1, 3, and 4, with Reviewer 3 specifically concluding that the revised manuscript was methodologically sound. We therefore did not perform additional heterogeneity analyses in the present revision.

**REVIEWER #3**

**Comment**: *Excellent work! All methodological concerns have been thoroughly addressed, with appropriate revisions and clarifications. The manuscript is now methodologically sound and satisfactory.*

**Response:** We sincerely thank the Reviewer for the careful reassessment and positive evaluation of the revised manuscript.

**REVIEWER #4**

**Comment:** *Thank you for addressing several of my comments and concerns. I appreciate the authors' efforts in revising the manuscript.*

**Response**: We thank the Reviewer for the thoughtful review and acknowledgment of our revisions.
