# Supplementary material for: Dual antiplatelet therapy duration after percutaneous coronary intervention with contemporary drug-eluting stents: A systematic review and network meta-analysis of randomized trials
Source: PLoS One. 2026 Sep 2;21(9):e0357462. doi: 10.1371/journal.pone.0357462 (PMC13537542; doi:10.1371/journal.pone.0357462)
Supplement: Peer review history

# Peer review: Decision Letter 0

**DOI:** 10.1371/journal.pone.0357462.r001

**Authors:** Haipeng Liu

10 Jul 2026

Dear Dr. Hlupeni,

Thank you for submitting your manuscript to PLOS ONE. After careful consideration, we feel that it has merit but does not fully meet PLOS ONE’s publication criteria as it currently stands. Therefore, we invite you to submit a revised version of the manuscript that addresses the points raised during the review process.

Please submit your revised manuscript by Sep 08 2026 11:59PM. If you will need more time than this to complete your revisions, please reply to this message or contact the journal office at plosone@plos.org. When you're ready to submit your revision, log on to https://www.editorialmanager.com/pone/ and select the 'Submissions Needing Revision' folder to locate your manuscript file.

- A letter that responds to each point raised by the academic editor and reviewer(s). You should upload this letter as a separate file labeled 'Response to Reviewers'.
- A marked-up copy of your manuscript that highlights changes made to the original version. You should upload this as a separate file labeled 'Revised Manuscript with Track Changes'.
- An unmarked version of your revised paper without tracked changes. You should upload this as a separate file labeled 'Manuscript'.

If you would like to make changes to your financial disclosure, please include your updated statement in your cover letter. Guidelines for resubmitting your figure files are available below the reviewer comments at the end of this letter.

If applicable, we recommend that you deposit your laboratory protocols in protocols.io to enhance the reproducibility of your results. Protocols.io assigns your protocol its own identifier (DOI) so that it can be cited independently in the future. For instructions see: https://journals.plos.org/plosone/s/submission-guidelines#loc-laboratory-protocols. Additionally, PLOS ONE offers an option for publishing peer-reviewed Lab Protocol articles, which describe protocols hosted on protocols.io. Read more information on sharing protocols at https://plos.org/protocols?utm_medium=editorial-email&utm_source=authorletters&utm_campaign=protocols.

As the corresponding author, your ORCID iD is verified in the submission system and will appear in the published article. PLOS supports the use of ORCID, and we encourage all coauthors to register for an ORCID iD and use it as well. Please encourage your coauthors to verify their ORCID iD within the submission system before final acceptance, as unverified ORCID iDs will not appear in the published article. *Only*  the individual author can complete the verification step; PLOS staff *cannot*  verify ORCID iDs on behalf of authors.

We look forward to receiving your revised manuscript.

Kind regards,

Haipeng Liu

Academic Editor

PLOS One

Journal Requirements:

When submitting your revision, we need you to address these additional requirements.

1. Please ensure that your manuscript meets PLOS ONE's style requirements, including those for file naming. The PLOS ONE style templates can be found at

https://journals.plos.org/plosone/s/file?id=wjVg/PLOSOne_formatting_sample_main_body.pdf and

https://journals.plos.org/plosone/s/file?id=ba62/PLOSOne_formatting_sample_title_authors_affiliations.pdf

2. Please update your submission to use the PLOS LaTeX template. The template and more information on our requirements for LaTeX submissions can be found at http://journals.plos.org/plosone/s/latex.

3. Please provide a complete Data Availability Statement in the submission form, ensuring you include all necessary access information or a reason for why you are unable to make your data freely accessible. If your research concerns only data provided within your submission, please write "All data are in the manuscript and/or supporting information files" as your Data Availability Statement.

4. We notice that your supplementary tables are included in the manuscript file. Please remove them and upload them with the file type 'Supporting Information'. Please ensure that each Supporting Information file has a legend listed in the manuscript after the references list.

5. If the reviewer comments include a recommendation to cite specific previously published works, please review and evaluate these publications to determine whether they are relevant and should be cited. There is no requirement to cite these works unless the editor has indicated otherwise.

[Note: HTML markup is below. Please do not edit.]

Reviewers' comments:

Reviewer's Responses to Questions

**Comments to the Author**

1. Is the manuscript technically sound, and do the data support the conclusions?

Reviewer #1: Yes

Reviewer #2: No

Reviewer #3: Partly

Reviewer #4: Yes

**********

2. Has the statistical analysis been performed appropriately and rigorously?

Reviewer #1: Yes

Reviewer #2: No

Reviewer #3: No

Reviewer #4: I Don't Know

**********

3. Have the authors made all data underlying the findings in their manuscript fully available?

The PLOS Data policy

Reviewer #1: Yes

Reviewer #2: Yes

Reviewer #3: No

Reviewer #4: Yes

**********

4. Is the manuscript presented in an intelligible fashion and written in standard English?

Reviewer #1: Yes

Reviewer #2: Yes

Reviewer #3: Yes

Reviewer #4: Yes

**********

Reviewer #1: This systematic review and NMA evaluates the optimal duration of DAPT after PCI with contemporary DES. The authors included 28 RCTs involving 84,325 patients and compared abbreviated DAPT strategies of 1, 3, and 6 months with the standard 12-month DAPT strategy. They concluded that abbreviated DAPT was not associated with a statistically significant increase in MACE compared with 12-month therapy. However, 1-month and 3-month DAPT were associated with a significant reduction in major bleeding. The authors suggest that 3-month DAPT may offer the most favorable balance between ischemic and bleeding outcomes. I have the following comments for the authors:

1-The manuscript stratifies studies according to DAPT duration, but the antiplatelet strategy after DAPT discontinuation varies substantially across trials. The authors should stratify the analyses according to the post-DAPT monotherapy strategy, when feasible, or explicitly state that the treatment groups combine heterogeneous post-DAPT antiplatelet regimens.

2-The authors should avoid language implying the superiority of 3-month DAPT for ischemic protection. This finding should be interpreted with caution and presented as exploratory, particularly given the limitations inherent to study-level NMA and the heterogeneity across trials.

3-The global inconsistency test showed a borderline p-value of 0.05. The authors state that “this inconsistency was resolved in the subgroup analysis stratified by study setting (multinational studies vs. single-country studies)”. However, the results of the analysis supporting this statement are not reported. The authors should provide these results either in the main text or in the Supplementary Appendix.

4-The authors rate the certainty of evidence as “high” for both MACE and major bleeding. Yet, given the heterogeneity of endpoint definitions, the use of study-level rather than patient-level data, and the borderline inconsistency signal, a “moderate” certainty rating for MACE may be more appropriate unless further sensitivity analyses support the robustness of the findings.

5-The authors should consider additional sensitivity analyses excluding studies at high risk of bias, and studies enrolling mixed ACS and stable CAD populations. Analyses separating ACS-only populations from stable CAD or mixed populations would be particularly informative given the potential differences observed in previous meta-analyses by clinical presentation (PMID: 39226909).

6-The Methods section defines standard 12-month DAPT as the comparator and abbreviated DAPT as the intervention. However, the bleeding network includes a node for DAPT >12 months, whereas this node does not appear to be included for MACE. The authors should explain the rationale for including this node for bleeding outcomes but not for ischemic outcomes.

7-The authors may consider expanding the Discussion by incorporating recent research articles on antithrombotic treatment after PCI. In particular, studies addressing de-escalation strategies (PMID: 39054275; PMID: 39415380) as well as long-term antiplatelet monotherapy (PMID: 41697985; PMID: 41211721) are highly relevant to the topic and should be discussed.

8-The conclusion should be rephrased, as the current wording is too generic for a population that likely includes patients with heterogeneous ischemic and bleeding risks. The authors should emphasize that the optimal DAPT duration should remain individualized according to clinical presentation, ischemic risk, bleeding risk, procedural complexity, and the planned post-DAPT antiplatelet strategy.

9-The title is informative but somewhat long. The authors should consider shortening it while preserving the key elements of the study design and research question.

Reviewer #2: The manuscript by et al, entitled: “Optimal duration of dual antiplatelet therapy after percutaneous coronary intervention with contemporary drug-eluting stents: A systematic review and network meta-analysis of 28 randomized trials” is a network meta-analysis comparing different DAPT regimens after PCI with new DES including 28 RCTs and >84000 patients.

Despite I recognize the authors did a lot of efforts, I am sorry to say that this study is not novel and does not add any significant news on the topic that has been explored by several previous meta-analyses. Additionally, there are crucial limitations shared with previous meta-analyses related to the mixture of different RCTs with different trials, designs, regimens, definitions, timing, etc and poor granularity of data by a study-level approach. Therefore results and conclusions have poor reliability and impact.

Reviewer #3: Major Comment 1

Problem: The authors assign a "high" certainty rating to the MACE outcome despite identifying marginal global inconsistency (p=0.05) and significant local inconsistency at the 3-month node (p=0.01 for 12m vs. 3m; p=0.007 for 1m vs. 3m). This rating is incongruent with GRADE and CINeMA frameworks, which require downgrading for inconsistency and imprecision when all primary efficacy confidence intervals span the null.

Recommendation: Downgrade the certainty of evidence for MACE to "Moderate" or "Low" and provide the complete node-splitting results in the main text or supplement to justify the reliability of the pooled estimates.

Major Comment 2

Problem: There is substantial heterogeneity in the primary efficacy endpoint (MACE) definitions across the 28 trials, with some including target vessel revascularization (TVR) and others restricted to "hard" clinical events [33, 41, 82–93]. This variability threatens the transitivity assumption, as indirect comparisons through a common 12-month comparator may be biased by the lack of exchangeability in outcome sensitivity.

Recommendation: Perform a sensitivity analysis restricted to trials using a standardized "hard" ischemic endpoint (e.g., death, MI, and stroke) to verify the robustness of the network estimates and rankings.

Major Comment 3

Problem: The conclusion that 3-month DAPT is the "optimal strategy" and "pragmatic default option" relies heavily on probabilistic SUCRA rankings rather than statistically significant differences in effect. Point estimates for all abbreviated durations versus 12-month DAPT are close to unity with widely overlapping confidence intervals, rendering rankings potentially unstable and clinically insignificant.

Recommendation: Revise the conclusion to state that abbreviated regimens offer comparable ischemic protection to 12-month DAPT, and clarify that the choice between 1-, 3-, and 6-month durations should be driven by the significant bleeding reductions rather than SUCRA-based efficacy hierarchies.

Major Comment 4

Problem: The manuscript does not include a formal assessment for publication bias or small-study effects despite the inclusion of 28 trials. In a network of this size, it is essential to demonstrate that the observed results for efficacy and safety are not skewed by the omission of small, non-significant trials.

Recommendation: Provide a comparison-adjusted funnel plot and a corresponding statistical test (e.g., Egger’s or Harbord’s test) to evaluate the potential impact of reporting bias on the network results.

Major Comment 5

Problem: The authors claim that inconsistency in the efficacy network was "resolved" through subgroup analysis by study setting (single-country vs. multi-country trials) but do not report the statistical tests to support this assertion. Without reporting the global and local inconsistency p-values within these specific subgroups, the statistical validity of the final model remains unverified.

Recommendation: Explicitly report the p-values for global and local inconsistency tests for each analyzed subgroup to demonstrate that the stratification effectively addressed the original transitivity violation.

Reviewer #4: Authors addresses an important and clinically relevant question, and the systematic review and network meta-analysis appear to be carefully conducted. The findings are generally consistent with the evidence supporting abbreviated DAPT after PCI with using DES.

However, I would encourage the authors to better clarify the novelty and clinical value of the present study. Several guidelines already recommend shorter DAPT durations than the traditional 12-month regimen, based on several large randomized trials. In this context, it is not entirely clear what additional knowledge this network meta-analysis provides beyond the existing evidence.

Overall, I believe the manuscript would be strengthened by a clearer explanation of how these findings add to the current evidence base and how they may influence clinical decision-making beyond the recommendations already reflected in contemporary guidelines.

**********

what does this mean?). If published, this will include your full peer review and any attached files.

If you choose “no”, your identity will remain anonymous but your review may still be made public.

**Do you want your identity to be public for this peer review?**  For information about this choice, including consent withdrawal, please see our Privacy Policy

Reviewer #1: No

Reviewer #2: No

Reviewer #3: No

Reviewer #4: No

**********

[NOTE: If reviewer comments were submitted as an attachment file, they will be attached to this email and accessible via the submission site. Please log into your account, locate the manuscript record, and check for the action link "View Attachments". If this link does not appear, there are no attachment files.]

To ensure your figures meet our technical requirements, please review our figure guidelines: https://journals.plos.org/plosone/s/figures

You may also use PLOS’s free figure tool, NAAS, to help you prepare publication quality figures: https://journals.plos.org/plosone/s/figures#loc-tools-for-figure-preparation.

NAAS will assess whether your figures meet our technical requirements by comparing each figure against our figure specifications.

# Author comment: Author response to Decision Letter 1

**DOI:** 10.1371/journal.pone.0357462.r002

20 Jul 2026

GENERAL RESPONSE

We thank the Editor and Reviewers for their thoughtful and constructive feedback. We have carefully revised the manuscript to address all comments. All changes have been incorporated into the revised manuscript and are highlighted in the tracked version. Below, we provide a point-by-point response.

REVIEWER #1

Comment 1: Post-DAPT monotherapy strategies vary across trials… stratify or clarify.

Response: We thank the reviewer for this important observation. We agree that post-DAPT antiplatelet strategies varied across trials and represent a potential source of clinical heterogeneity. Accordingly, we have explicitly acknowledged this heterogeneity in the Methods and Limitations sections, clarifying that treatment groups may include varying post-DAPT monotherapy regimens (aspirin vs. P2Y12 inhibitor).

Given the limited reporting granularity across trials, formal stratification by post-DAPT regimen was not feasible.

Comment 2: Avoid implying superiority of 3-month DAPT.

Response: We agree and have revised the manuscript accordingly.

• All language implying superiority has been removed.

• SUCRA findings are now consistently described as probabilistic and exploratory.

Comment 3: Provide subgroup inconsistency results.

Response: We have now explicitly reported these results in both the Results section and Supplementary Material: Multicountry trials: p = 0.94, Single-country trials: p = 0.34. Additionally, full node-splitting results have been included in Supplementary Table S2

Comment 4: GRADE rating too high for MACE.

Response: We agree and have revised the certainty of evidence. MACE downgraded from High to Moderate. Reasons included inconsistency (borderline global inconsistency) and imprecision (confidence intervals crossing unity)

Comment 5: Additional sensitivity analyses (ACS vs mixed populations).

Response: We thank the reviewer for this valuable suggestion. We have now performed and included:

• ACS-only analysis vs Mixed/stable population analysis

Results presented in Main Results section, Supplementary Table S3 and Supplementary Figure S2. Findings were consistent with the primary analysis.

Comment 6: >12-month node discrepancy between networks.

Response: We have clarified this in the Results section. The >12-month node was not included in the MACE network because trials evaluating extended DAPT did not report MACE as a standalone endpoint.

Comment 7: Include recent literature (de-escalation, monotherapy).

Response: We have expanded the Discussion and added relevant recent studies (PMID: 39054275; PMID: 39415380).

Comment 8: Conclusion too generic.

Response: We agree and have revised the conclusion to emphasize:

• Individualization of DAPT duration

• Role of ischemic vs bleeding risk

• Procedural complexity

• Post-DAPT strategy

Comment 9: Title too long.

Response: We have shortened the title while preserving key elements.

REVIEWER #2

Comment: Study is not novel and lacks impact.

Response: We respectfully disagree and have revised the manuscript to better clarify its novelty.

Key contributions of our study:

• Direct comparison across multiple abbreviated DAPT durations (1-, 3-, 6-month). This has not been addressed in prior pairwise meta-analyses

• Separation of ischemic and bleeding outcomes: Prior studies relied heavily on composite endpoints (e.g., NACE)

• Granular comparative framework: Allows clinicians to differentiate between abbreviated strategies

We have strengthened the Introduction and Discussion by including clarifications such as “While current guidelines and prior meta-analyses support DAPT abbreviation, they do not provide direct comparative insight across abbreviated strategies. Our network meta-analysis addresses this gap…”

REVIEWER #3

Major Comment 1: GRADE inconsistency

Response: Addressed as above, MACE downgraded to Moderate and Node-splitting results added to Supplementary information

Major Comment 2: MACE heterogeneity threatens transitivity

Response: We agree and have performed additional sensitivity analysis: Restricted to hard ischemic endpoints (death, MI, stroke). No significant differences observed and findings were consistent with primary analysis, though less precise due to fewer studies in other subgroups. We added these additional analyses to Results section and Supplementary Table S3

Major Comment 3: Over-reliance on SUCRA

Response: We agree and revised all relevant sections. We removed language implying “optimal strategy” and emphasized no non-significant differences in MACE outcomes and the fact that SUCRA ranking are probabilistic.

Major Comment 4: Publication bias not assessed

Response: We have now included comparison-adjusted funnel plots (Supplementary Figure S1) and Egger’s regression test, MACE: p = 0.63, Bleeding: p = 0.20

Major Comment 5: Inconsistency resolution not supported

Response: We have now explicitly reported global inconsistency p-values by subgroup: Multicountry: 0.94 and Single-country: 0.34. Supporting data included in Supplementary materials.

REVIEWER #4

Comment: Clarify novelty and clinical value

Response: We appreciate this important point and have strengthened the manuscript accordingly.

We now emphasize that our study moves beyond “whether to shorten DAPT” to “how abbreviated strategies compare with one another”. We added clarifications such as “Our analysis provides comparative insight across commonly used abbreviated DAPT strategies, addressing a key gap not resolved by existing guidelines or prior meta-analyses.”

Attachment Submitted filename: Response to Reviewers.docx

# Peer review: Decision Letter 1

**DOI:** 10.1371/journal.pone.0357462.r003

**Authors:** Haipeng Liu

17 Aug 2026

Dear Dr. Hlupeni,

Thank you for submitting your manuscript to PLOS ONE. After careful consideration, we feel that it has merit but does not fully meet PLOS ONE’s publication criteria as it currently stands. Therefore, we invite you to submit a revised version of the manuscript that addresses the points raised during the review process.

Please submit your revised manuscript by Sep 16 2026 11:59PM. If you will need more time than this to complete your revisions, please reply to this message or contact the journal office at plosone@plos.org. When you're ready to submit your revision, log on to https://www.editorialmanager.com/pone/ and select the 'Submissions Needing Revision' folder to locate your manuscript file.

- A letter that responds to each point raised by the academic editor and reviewer(s). You should upload this letter as a separate file labeled 'Response to Reviewers'.
- A marked-up copy of your manuscript that highlights changes made to the original version. You should upload this as a separate file labeled 'Revised Manuscript with Track Changes'.
- An unmarked version of your revised paper without tracked changes. You should upload this as a separate file labeled 'Manuscript'.

If you would like to make changes to your financial disclosure, please include your updated statement in your cover letter. Guidelines for resubmitting your figure files are available below the reviewer comments at the end of this letter.

If applicable, we recommend that you deposit your laboratory protocols in protocols.io to enhance the reproducibility of your results. Protocols.io assigns your protocol its own identifier (DOI) so that it can be cited independently in the future. For instructions see: https://journals.plos.org/plosone/s/submission-guidelines#loc-laboratory-protocols. Additionally, PLOS ONE offers an option for publishing peer-reviewed Lab Protocol articles, which describe protocols hosted on protocols.io. Read more information on sharing protocols at https://plos.org/protocols?utm_medium=editorial-email&utm_source=authorletters&utm_campaign=protocols.

As the corresponding author, your ORCID iD is verified in the submission system and will appear in the published article. PLOS supports the use of ORCID, and we encourage all coauthors to register for an ORCID iD and use it as well. Please encourage your coauthors to verify their ORCID iD within the submission system before final acceptance, as unverified ORCID iDs will not appear in the published article. *Only*  the individual author can complete the verification step; PLOS staff *cannot*  verify ORCID iDs on behalf of authors.

We look forward to receiving your revised manuscript.

Kind regards,

Haipeng Liu

Academic Editor

PLOS One

Journal Requirements:

If the reviewer comments include a recommendation to cite specific previously published works, please review and evaluate these publications to determine whether they are relevant and should be cited. There is no requirement to cite these works unless the editor has indicated otherwise.

Please review your reference list to ensure that it is complete and correct. If you have cited papers that have been retracted, please include the rationale for doing so in the manuscript text, or remove these references and replace them with relevant current references. Any changes to the reference list should be mentioned in the rebuttal letter that accompanies your revised manuscript. If you need to cite a retracted article, indicate the article’s retracted status in the References list and also include a citation and full reference for the retraction notice.

Additional Editor Comments:

Review 2 pointed out some important trials are not included. Please double check the literature search strategy. Regarding the inclusion of some latest studies, I suggest to do an updated search. They can be included in your discussion if the publication date is later than the deadline of your literature search. The heterogeneity analysis should also be improved.

[Note: HTML markup is below. Please do not edit.]

Reviewers' comments:

Reviewer's Responses to Questions

**Comments to the Author**

Reviewer #1: All comments have been addressed

Reviewer #2: (No Response)

Reviewer #3: All comments have been addressed

Reviewer #4: All comments have been addressed

**********

2. Is the manuscript technically sound, and do the data support the conclusions?

Reviewer #1: Yes

Reviewer #2: No

Reviewer #3: Yes

Reviewer #4: Yes

**********

3. Has the statistical analysis been performed appropriately and rigorously?

Reviewer #1: Yes

Reviewer #2: No

Reviewer #3: Yes

Reviewer #4: Yes

**********

4. Have the authors made all data underlying the findings in their manuscript fully available?

The PLOS Data policy

Reviewer #1: Yes

Reviewer #2: No

Reviewer #3: Yes

Reviewer #4: Yes

**********

5. Is the manuscript presented in an intelligible fashion and written in standard English?

Reviewer #1: Yes

Reviewer #2: No

Reviewer #3: Yes

Reviewer #4: Yes

**********

Reviewer #1: The manuscript has been substantially revised in accordance with the Reviewers' requests. I have no further comments for the authors.

Reviewer #2: I am sorry to disagree and to respectfully say that I continue to see poor novelty. The topic is overbooked and there are hundreds of publications including trials, meta-analyses, expert reviews, consensus documents and international guidelines on the “optimal DAPT”. Yet, the review is not properly updated given that few weeks ago the DAPT MVD has been published on the NEJM. I also have concerns in terms of studies included, for example I see EXCELLENT, RESET, PRODIGY and DAPT trials missing, but these are only examples because honestly I continue to see impossibility to properly manage the huge heterogeneity across trials designs, definitions, etc. and therefore absence of sufficient quality and novelty to advance our current knowledge.

Reviewer #3: Excellent work! All methodological concerns have been thoroughly addressed, with appropriate revisions and clarifications. The manuscript is now methodologically sound and satisfactory.

Reviewer #4: Thank you for addressing several of my comments and concerns. I appreciate the authors' efforts in revising the manuscript.

**********

what does this mean?). If published, this will include your full peer review and any attached files.

If you choose “no”, your identity will remain anonymous but your review may still be made public.

**Do you want your identity to be public for this peer review?**  For information about this choice, including consent withdrawal, please see our Privacy Policy

Reviewer #1: No

Reviewer #2: No

Reviewer #3: No

Reviewer #4: No

**********

[NOTE: If reviewer comments were submitted as an attachment file, they will be attached to this email and accessible via the submission site. Please log into your account, locate the manuscript record, and check for the action link "View Attachments". If this link does not appear, there are no attachment files.]

To ensure your figures meet our technical requirements, please review our figure guidelines: https://journals.plos.org/plosone/s/figures

You may also use PLOS’s free figure tool, NAAS, to help you prepare publication quality figures: https://journals.plos.org/plosone/s/figures#loc-tools-for-figure-preparation.

NAAS will assess whether your figures meet our technical requirements by comparing each figure against our figure specifications.

# Author comment: Author response to Decision Letter 2

**DOI:** 10.1371/journal.pone.0357462.r004

17 Aug 2026

Manuscript ID: PONE-D-26-23217R1

Title: Dual antiplatelet therapy duration after percutaneous coronary intervention with contemporary drug-eluting stents: A systematic review and network meta-analysis of randomized trials

Dear Dr. Liu and Reviewers,

Thank you for your careful review of our manuscript. We appreciate that Reviewers 1, 3, and 4 considered their previous concerns adequately addressed. We have carefully considered the remaining comments from Reviewer 2 and the Academic Editor and have made additional revisions as detailed below.

ACADEMIC EDITOR

Comment: Review 2 pointed out some important trials are not included. Please double check the literature search strategy. Regarding the inclusion of some latest studies, I suggest to do an updated search. They can be included in your discussion if the publication date is later than the deadline of your literature search. The heterogeneity analysis should also be improved.

Response: Thank you for these recommendations. We rechecked our literature search and eligibility assessment and confirmed that the trials specifically identified by Reviewer 2 were captured by our search but excluded according to predefined eligibility criteria. These exclusions were already explicitly reported in the manuscript (Lines 137–140). We also reviewed recently published evidence and have incorporated the DAPT-MVD trial into the Discussion as important contemporary evidence published after our prespecified search period.

Regarding heterogeneity, this concern had been extensively addressed in Revision 1 through global and local inconsistency assessments and additional sensitivity analyses according to ACS presentation and MACE endpoint definition, including an analysis restricted to hard ischemic endpoints. Complete node-splitting results were also provided in the Supporting Information, and the certainty of evidence for MACE was downgraded to moderate because of inconsistency and imprecision. These revisions were considered satisfactory by Reviewers 1, 3, and 4, with Reviewer 3 specifically concluding that the manuscript was methodologically sound. Accordingly, no additional heterogeneity analyses were performed in the present revision.

REVIEWER #1

Comment: The manuscript has been substantially revised in accordance with the Reviewers' requests. I have no further comments for the authors.

Response: We thank the Reviewer for the careful review and positive assessment of our revised manuscript.

REVIEWER #2

Comment: I continue to see poor novelty. The topic is overbooked and there are hundreds of publications including trials, meta-analyses, expert reviews, consensus documents and international guidelines on the “optimal DAPT”.

Response: We appreciate the Reviewer’s concern and agree that the broad question of optimal DAPT duration has been extensively studied. However, the present study does not seek to re-examine the broad question of “optimal DAPT duration” across abbreviated, conventional, and extended treatment strategies. Rather, our research question is specifically focused on DAPT abbreviation (<12 months): how the commonly used 1-, 3-, and 6-month abbreviated regimens compare with one another and whether the available evidence identifies a clinically meaningful threshold for abbreviation in the contemporary DES era. This distinction is important because, although abbreviated DAPT is increasingly supported by randomized trials and current guidelines, the individual abbreviated strategies have predominantly been evaluated against conventional-duration therapy rather than against one another. By integrating direct and indirect evidence, our network meta-analysis provides simultaneous comparison of these abbreviated strategies while separately evaluating ischemic and bleeding outcomes. Our prior revision had further emphasized this specific research question in the Introduction and Discussion to avoid conflating DAPT abbreviation with the broader literature on optimal DAPT duration.

Comment: The review is not properly updated given that few weeks ago the DAPT MVD has been published on the NEJM.

Response: We thank the Reviewer for highlighting this important recent trial. DAPT-MVD was published after our prespecified literature-search period and has now been incorporated into the Discussion. DAPT-MVD evaluates extended DAPT beyond 12 months in selected patients with multivessel coronary disease and therefore addresses a different therapeutic question from the principal focus of our study, which is the comparative performance and threshold of abbreviated (<12-month) DAPT strategies.

Comment: I also have concerns in terms of studies included, for example I see EXCELLENT, RESET, PRODIGY and DAPT trials missing...

Response: We appreciate the opportunity to clarify this point. These trials were not missed by our literature search; they were identified, evaluated, and excluded according to our predefined eligibility criteria. These exclusions were already explicitly reported in the manuscript (Lines 137-140). Specifically, PRODIGY, RESET, and EXCELLENT are cited as references 48–50, respectively, and were excluded because of substantial first-generation stent use, inconsistent with our prespecified contemporary-DES eligibility criterion. The DAPT trial evaluated extended therapy without an abbreviated (<12-month) DAPT arm and therefore did not meet the eligibility criteria for the principal research question. We rechecked the literature search and confirmed these exclusions.

Comment: I continue to see impossibility to properly manage the huge heterogeneity across trials designs, definitions, etc. and therefore absence of sufficient quality and novelty to advance our current knowledge.

Response: We agree that heterogeneity across DAPT trials is an important consideration. This concern was extensively addressed in Revision 1 through global and local inconsistency testing and additional sensitivity analyses according to ACS presentation and MACE endpoint definition, including an analysis restricted to standardized hard ischemic endpoints (death, myocardial infarction, and stroke). Complete node-splitting results were provided in the Supporting Information, and the certainty of evidence for MACE was downgraded to moderate because of inconsistency and imprecision. These methodological revisions were considered satisfactory by Reviewers 1, 3, and 4, with Reviewer 3 specifically concluding that the revised manuscript was methodologically sound. We therefore did not perform additional heterogeneity analyses in the present revision.

REVIEWER #3

Comment: Excellent work! All methodological concerns have been thoroughly addressed, with appropriate revisions and clarifications. The manuscript is now methodologically sound and satisfactory.

Response: We sincerely thank the Reviewer for the careful reassessment and positive evaluation of the revised manuscript.

REVIEWER #4

Comment: Thank you for addressing several of my comments and concerns. I appreciate the authors' efforts in revising the manuscript.

Response: We thank the Reviewer for the thoughtful review and acknowledgment of our revisions.

Attachment Submitted filename: Response_to_Reviewers_auresp_2.docx

# Editor report: Decision Letter 2

**DOI:** 10.1371/journal.pone.0357462.r005

**Authors:** Haipeng Liu

18 Aug 2026

Dual antiplatelet therapy duration after percutaneous coronary intervention with contemporary drug-eluting stents: A systematic review and network meta-analysis of randomized trials

PONE-D-26-23217R2

Dear Dr. Hlupeni,

We’re pleased to inform you that your manuscript has been judged scientifically suitable for publication and will be formally accepted for publication once it meets all outstanding technical requirements.

Within one week, you’ll receive an e-mail detailing the required amendments. When these have been addressed, you’ll receive a formal acceptance letter and your manuscript will be scheduled for publication.

An invoice will be generated when your article is formally accepted. Please note, if your institution has a publishing partnership with PLOS and your article meets the relevant criteria, all or part of your publication costs will be covered. Please make sure your user information is up-to-date by logging into Editorial Manager at Editorial Manager® and clicking the ‘Update My Information' link at the top of the page. For questions related to billing, please contact billing support.

If your institution or institutions have a press office, please notify them about your upcoming paper to help maximize its impact. If they’ll be preparing press materials, please inform our press team as soon as possible -- no later than 48 hours after receiving the formal acceptance. Your manuscript will remain under strict press embargo until 2 pm Eastern Time on the date of publication. For more information, please contact onepress@plos.org.

Kind regards,

Haipeng Liu

Academic Editor

PLOS One

Additional Editor Comments (optional):

Reviewers' comments:

# Editor report: Acceptance letter

**DOI:** 10.1371/journal.pone.0357462.r006

**Authors:** Haipeng Liu

PONE-D-26-23217R2

PLOS One

Dear Dr. Hlupeni,

I'm pleased to inform you that your manuscript has been deemed suitable for publication in PLOS One. Congratulations! Your manuscript is now being handed over to our production team.

At this stage, our production department will prepare your paper for publication. This includes ensuring the following:

* All references, tables, and figures are properly cited

* All relevant supporting information is included in the manuscript submission,

* There are no issues that prevent the paper from being properly typeset

You will receive further instructions from the production team, including instructions on how to review your proof when it is ready. Please keep in mind that we are working through a large volume of accepted articles, so please give us a few days to review your paper and let you know the next and final steps.

Lastly, if your institution or institutions have a press office, please let them know about your upcoming paper now to help maximize its impact. If they'll be preparing press materials, please inform our press team within the next 48 hours. Your manuscript will remain under strict press embargo until 2 pm Eastern Time on the date of publication. For more information, please contact onepress@plos.org.

You will receive an invoice from PLOS for your publication fee after your manuscript has reached the completed accept phase. If you receive an email requesting payment before acceptance or for any other service, this may be a phishing scheme. Learn how to identify phishing emails and protect your accounts at https://explore.plos.org/phishing.

If we can help with anything else, please email us at customercare@plos.org.

Thank you for submitting your work to PLOS One and supporting open access.

Kind regards,

PLOS One Editorial Office Staff

on behalf of

Dr. Haipeng Liu

Academic Editor

PLOS One
